# Supplementary figures and images for: The impact of ultrasound-based antenatal screening strategies to detect vasa praevia in the United Kingdom: An exploratory study using decision analytic modelling methods
Source: PLoS One. 2022 Dec 20;17(12):e0279229. doi: 10.1371/journal.pone.0279229 (PMC9767376; doi:10.1371/journal.pone.0279229)

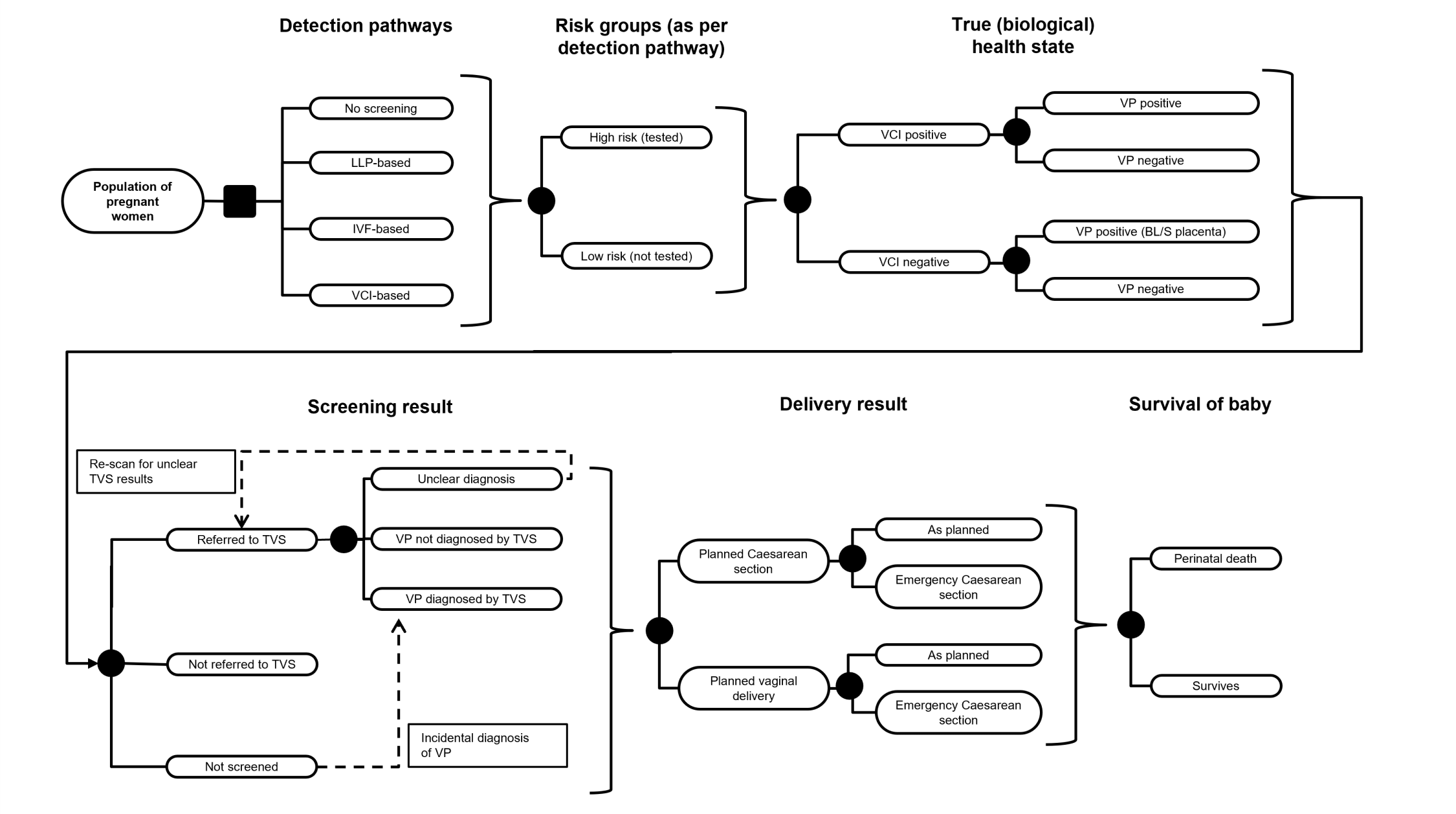

Supplement: S1 Fig — (TIF) [file pone.0279229.s001.tif]
