# Supplementary material for: The impact of ultrasound-based antenatal screening strategies to detect vasa praevia in the United Kingdom: An exploratory study using decision analytic modelling methods
Source: PLoS One. 2022 Dec 20;17(12):e0279229. doi: 10.1371/journal.pone.0279229 (PMC9767376; doi:10.1371/journal.pone.0279229)
Supplement: S1 Table — (DOCX) [file pone.0279229.s002.docx]

S1 Table: Full list of inputs, including rationale and references

| **Input** | **Value ^a^** | **Rationale** | **Reference** |
| --- | --- | --- | --- |
| **Population-level model input** | | | |
| The total number of pregnancies in the UK population per year | 862,785 | Official UK statistics, providing accurate, population-level data | ONS [[1](#_ENREF_1)] |
| **VP risk factor prevalence** | | | |
| Prevalence of IVF (general population) | 1.60% | Appropriate literature value (based on applicability and quality of the study) identified through targeted searches | Ebbing 2013 [[2](#_ENREF_2)] |
| Prevalence of LLP (general population) | 10.00% | Based on expert opinion regarding the estimated prevalence of LLP as experienced in UK clinical practice | Expert opinion |
| Prevalence of LLP (IVF pregnancies) | 56.00% | Obtained by multiplying the estimate for the prevalence of LLP with the OR for placenta previa amongst IVF pregnancies identified in a large Norwegian population-based study | Expert opinion /  Romundstad 2006 [[3](#_ENREF_3)] (for odds ratio of LLP in IVF) |
| **Incidence of VCI** | | | |
| General population | 1.50%  (95% CI: 1.49; 1.55)* | Appropriate literature value (based on applicability and quality of the study) identified through targeted searches | Ebbing 2013 [[2](#_ENREF_2)] |
| IVF pregnancies | 3.70%  (95% CI: 3.31; 4.04)* | Appropriate literature value (based on applicability and quality of the study) identified through targeted searches | Ebbing 2013 [[2](#_ENREF_2)] |
| LLP pregnancies | 2.80%  (95% CI: 0.95; 7.85)* | Appropriate literature value (based on applicability and quality of the study) identified through targeted searches | Suzuki 2015 [[4](#_ENREF_4)] |
| VP pregnancies | 80.00%  (range: 40.00–100.00) | Average value identified in the UK NSC review, validated through expert opinion | UK NSC 2017 [[5](#_ENREF_5)] / Expert opinion |
| **Incidence of VP** | | | |
| General population | 0.03%  (95% CI: 0.01; 0.09)* | Average value identified in the UK NSC review, in alignment with expert opinion | UK NSC 2017 [[5](#_ENREF_5)] |
| IVF pregnancies | 0.34%  (95% CI: 0.13; 0.87)* | Appropriate literature value (based on applicability and quality of the study) identified through targeted searches | Schachter 2002 [[6](#_ENREF_6)] |
| LLP pregnancies | 0.52%  (95% CI: 0.20; 1.33)* | Appropriate literature value (based on applicability and quality of the study) identified through targeted searches | Rosenberg 2011 [[7](#_ENREF_7)] |
| **Incidence of BL/S placenta** | | | |
| General population | 3.08%  (95% CI: 2.16; 4.36)* | Appropriate literature value (based on applicability and quality of the study) identified through targeted searches | Manikanta Reddy 2013 [[8](#_ENREF_8)] |
| VP pregnancies | 20.00%  (range: 00.00–60.00) | Assumption that the remaining VP cases that do not involve VCI (see above) would be amongst BL/S placenta pregnancies | Assumption |
| **LLP diagnostic test accuracy** | | | |
| TAS for LLP (sensitivity) | 99.00%  (range: 95.00–100.00) | Appropriate literature value (based on applicability and quality of the study) identified through targeted searches | Cipriano 2010 [[9](#_ENREF_9)] |
| TAS for LLP (specificity) | 99.50%  (range: 99.00–100.00) | Appropriate literature value (based on applicability and quality of the study) identified through targeted searches | Cipriano 2010 [[9](#_ENREF_9)] |
| **VCI and BL/S placenta diagnostic test accuracy** | | | |
| TAS for VCI (sensitivity) | 99.16%  (95% CI: 98.27; 99.59)* | Appropriate literature value (based on applicability and quality of the study) identified through targeted searches | Sepulveda 2003 [[10](#_ENREF_10)] |
| TAS for BL/S placenta (sensitivity) | 75.00%  (range: 65.00–85.00) | Appropriate literature value (based on applicability and quality of the study) identified through targeted searches | Cipriano 2010 [[9](#_ENREF_9)] |
| TAS for BL/S placenta (specificity) | 99.50%  (range: 99.00–100.00) | Appropriate literature value (based on applicability and quality of the study) identified through targeted searches | Cipriano 2010 [[9](#_ENREF_9)] |
| **VP diagnostic test accuracy** | | | |
| TAS for VP (sensitivity) | 86.67%  (95% CI: 62.21; 96.26)* | Appropriate literature value (based on applicability and quality of the study) identified through targeted searches | Catanzarite 2001 [[11](#_ENREF_11)] |
| TAS for VP (specificity) | 98.00%  (95% CI: 93.00; 99.45)* | Assumed to be slightly lower than the specificity via TVS (see below) | Assumption |
| TVS for VP (sensitivity) | 96.55%  (95% CI: 88.27; 99.05)* | Appropriate literature value (based on applicability and quality of the study) identified through targeted searches | Bronsteen 2013 [[12](#_ENREF_12)] |
| TVS for VP (specificity) | 99.90%  (95% CI: 99.44; 99.98)* | Appropriate literature value (based on applicability and quality of the study) identified through targeted searches | Catanzarite 2001 [[11](#_ENREF_11)] |
| **Proportion of screening opt-outs (at 20 weeks)** | | | |
| General population | 0.01%  (95% CI: 0.00; 0.06)* | Based on expert opinion with regards to UK clinical practice | Expert opinion |
| IVF pregnancies | 0.01%  (95% CI: 0.00; 0.06)* | The proportion of opt-outs expected in IVF and LLP pregnancies was assumed to be equal to that of the general population. A conservative assumption, given that these groups are at higher risk of complications than the general population, meaning the mother may be less likely to decline testing | Assumption |
| LLP pregnancies | 0.01%  (95% CI: 0.00; 0.06)* |  |  |
| **Other diagnostic inputs** | | | |
| Unclear diagnosis from TVS for VP | 0.10%  (95% CI: 0.02; 0.56)* | Assumed to be a comparatively low rate of occurrence | Assumption |
| Incidental diagnosis of VP in LLP pregnancies (no screening pathway) | 6.00%  (95% CI: 4.69; 7.50)* | Assumed value, resulting in a number of antenatally detected VP pregnancies similar to what has been reported in UK clinical practice [[13](#_ENREF_13)] | Assumption |
| Incidental diagnosis of VP in the not screened cohort | 0.10%  (95% CI: 0.02; 0.56)* | Assumed to be a comparatively low rate of occurrence | Assumption |
| **Probability of planned Caesarean section** | | | |
| VP not diagnosed - general population | 12.00%  (95% CI: 11.92; 12.08)* | Official UK-specific statistics | NHS Maternity Statistics 2016-2017 [[14](#_ENREF_14)] |
| VP not diagnosed - IVF | 12.00%  (95% CI: 11.92; 12.08)* | The likelihood of planned Caesarean section is assumed to be similar for undiagnosed VP cases across all pregnancies, as a Caesarean section would not be recommended in relation to IVF or LLP alone | Assumption |
| VP not diagnosed - LLP | 12.00%  (95% CI: 11.92; 12.08)* |  |  |
| VP diagnosed | 100.00% | All diagnosed VP cases should undergo a planned Caesarean section, as recommended in the recent RCOG guidelines | Jauniaux (RCOG) 2018 [[15](#_ENREF_15)] |
| **Probability of emergency Caesarean section** | | | |
| General population | 15.50%  (95% CI: 15.41; 15.59)* | Official UK-specific statistics | NHS Maternity Statistics 2016-2017 [[14](#_ENREF_14)] |
| IVF pregnancy | 15.50%  (95% CI: 15.41; 15.59)* | The likelihood of emergency Caesarean section is assumed to be similar for IVF and LLP pregnancies without VP, and other pregnancies in the general population, as IVF and LLP are not associated with an increased risk of emergency Caesarean section | Assumption |
| LLP pregnancy | 15.50%  (95% CI: 15.41; 15.59)* |  |  |
| VP pregnancy (diagnosed) | 23.00%  (95% CI: 13.00, 36.00) | Informed by the MA of six studies | UK NSC / Costello Medical 2018 [[16](#_ENREF_16)] |
| VP pregnancy (undiagnosed) | 68.00%  (95% CI: 59.00, 76.00) | Informed by the MA of three studies | UK NSC / Costello Medical 2018 [[16](#_ENREF_16)] |
| VCI pregnancy | 14.00%  (95% CI: 15.00, 24.00) | Informed by the MA of 11 studies | UK NSC / Costello Medical 2018 [[16](#_ENREF_16)] |
| **Probability of perinatal mortality by birth method and type of pregnancy** | | | |
| Planned Caesarean section | | | |
| General population | 1.10%  (95% CI: 1.04; 1.16)* | Appropriate literature value (based on applicability and quality of the study) identified through targeted searches | Cipriano 2010 [[9](#_ENREF_9)] |
| IVF pregnancy | 1.10%  (95% CI: 1.04; 1.16)* | The mortality associated with planned Caesarean sections is assumed to be similar for non-VP cases across all risk groups. This assumes that LLP and IVF do not affect the risk of perinatal death in pregnancies that undergo planned Caesarean section | Assumed |
| LLP pregnancy | 1.10%  (95% CI: 1.04; 1.16)* |  | Assumed |
| VP pregnancy (diagnosed) | 3.00%  (95% CI: 1.00, 7.00) | Informed by the MA of six studies | UK NSC / Costello Medical 2018 [[16](#_ENREF_16)] |
| VCI pregnancy | 2.00%  (95% CI: 1.00, 4.00) | Informed by the MA of ten studies | UK NSC / Costello Medical 2018 [[16](#_ENREF_16)] |
| Emergency Caesarean section | | | |
| General population | 3.20%  (95% CI: 3.11; 3.30)* | Appropriate literature value (based on applicability and quality of the study) identified through targeted searches. The value represents late fetal or neonatal mortality leading to early Caesarean delivery, assumed to be equivalent to emergency Caesarean section | Cipriano 2010 [[9](#_ENREF_9)] |
| IVF pregnancy | 3.20%  (95% CI: 3.11; 3.30)* | The mortality associated with emergency Caesarean sections is assumed to be similar for non-VP cases across all risk groups. This assumes that LLP and IVF do not affect the risk of perinatal death in pregnancies that undergo emergency Caesarean section | Assumed |
| LLP pregnancy | 3.20%  (95% CI: 3.11; 3.30)* |  | Assumed |
| VP pregnancy (diagnosed) | 3.00%  (95% CI: 1.00, 7.00) | Informed by the MA of six studies | UK NSC / Costello Medical 2018 [[16](#_ENREF_16)] |
| VP pregnancy (undiagnosed) | 54.00%  (95% CI: 41.79; 65.69)* | Appropriate literature value (based on applicability and quality of the study) identified through the SLR (detailed in the Supplementary Appendix 1) | Oyelese 2004 [[17](#_ENREF_17)] |
| VCI pregnancy | 2.00%  (95% CI: 1.00, 4.00) | Informed by the MA of ten studies | UK NSC / Costello Medical 2018 [[16](#_ENREF_16)] |
| Vaginal delivery | | | |
| General population | 0.70%  (95% CI: 0.68; 0.72)* | Appropriate literature value (based on applicability and quality of the study) identified through targeted searches | Ebbing 2013 [[2](#_ENREF_2)] |
| IVF pregnancy | 0.70%  (95% CI: 0.68; 0.72)* | The mortality associated with vaginal delivery is assumed to be similar for non-VP cases across all risk groups. This assumes that LLP and IVF do not affect the risk of perinatal death in pregnancies that vaginal delivery | Assumption |
| LLP pregnancy | 0.70%  (95% CI: 0.68; 0.72)* |  |  |
| VP pregnancy (undiagnosed) | 69.20%  (95% CI: 50.68; 83.09)* | Appropriate literature value (based on applicability and quality of the study) identified through the SLR (detailed in the Supplementary Appendix 1) | Oyelese 2004 [[17](#_ENREF_17)] |
| VCI pregnancy | 2.00%  (95% CI: 1.00, 4.00) | Informed by the MA of ten studies | UK NSC / Costello Medical 2018 [[16](#_ENREF_16)] |

^a^ Confidence intervals indicated by an asterisk have been calculated, in the absence of published variance data, using the Wilson score interval method; values without listed range data have not been varied independently as part of the sensitivity analyses.

**Abbreviations:** BL/S, bilobed or succenturiate; CI, confidence interval; IVF, in vitro fertilisation; LLP, low-lying placenta; MA, meta-analysis; NHS, National Health Service; ONS, Office of National Statistics; OR, odds ratio; RCOG, Royal College of Obstetricians and Gynaecologists; SLR, systematic literature review; UK NSC, United Kingdom National Screening Committee; VCI, velamentous cord insertion; VP, vasa praevia.

**References**

1. Office for National Statistics. Conceptions in England and Wales: 2016 2018 [25th May 2018]. Available from: <https://www.ons.gov.uk/peoplepopulationandcommunity/birthsdeathsandmarriages/conceptionandfertilityrates/bulletins/conceptionstatistics/2016>.

2. Ebbing C, Kiserud T, Johnsen SL, Albrechtsen S, Rasmussen S. Prevalence, risk factors and outcomes of velamentous and marginal cord insertions: a population-based study of 634,741 pregnancies. PloS one. 2013;8(7):e70380. Epub 2013/08/13. doi: 10.1371/journal.pone.0070380. PubMed PMID: 23936197; PubMed Central PMCID: PMCPMC3728211.

3. Romundstad LB, Romundstad PR, Sunde A, von During V, Skjaerven R, Vatten LJ. Increased risk of placenta previa in pregnancies following IVF/ICSI; a comparison of ART and non-ART pregnancies in the same mother. Human reproduction (Oxford, England). 2006;21(9):2353-8. Epub 2006/05/27. doi: 10.1093/humrep/del153. PubMed PMID: 16728419.

4. Suzuki S, Kato M. Clinical Significance of Pregnancies Complicated by Velamentous Umbilical Cord Insertion Associated With Other Umbilical Cord/Placental Abnormalities. Journal of clinical medicine research. 2015;7(11):853-6. Epub 2015/10/23. doi: 10.14740/jocmr2310w. PubMed PMID: 26491497; PubMed Central PMCID: PMCPMC4596266.

5. UK National Screening Committee. Screening for vasa praevia in the second trimester of pregnacy - external review against programme appraisal criteria for the UK National Screening Committee (UK NSC). 2017.

6. Schachter M, Tovbin Y, Arieli S, Friedler S, Ron-El R, Sherman D. In vitro fertilization is a risk factor for vasa previa. Fertility and sterility. 2002;78(3):642-3. Epub 2002/09/07. PubMed PMID: 12215350.

7. Rosenberg T, Pariente G, Sergienko R, Wiznitzer A, Sheiner E. Critical analysis of risk factors and outcome of placenta previa. Archives of gynecology and obstetrics. 2011;284(1):47-51. Epub 2010/07/24. doi: 10.1007/s00404-010-1598-7. PubMed PMID: 20652281.

8. Manikanta Reddy. V, Senthil Kumar. S, Sanjeeva Reddy. N. Prevalence and Pattern of Abnormalities Occurring in Placenta and Umbilical Cord. International Journal of Medical Research and Health Sciences. 2013;2(4):935-40.

9. Cipriano LE, Barth Jr WH, Zaric GS. The cost-effectiveness of targeted or universal screening for vasa praevia at 18–20 weeks of gestation in Ontario. BJOG: An International Journal of Obstetrics & Gynaecology. 2010;117(9):1108-18. doi: 10.1111/j.1471-0528.2010.02621.x.

10. Sepulveda W, Rojas I, Robert JA, Schnapp C, Alcalde JL. Prenatal detection of velamentous insertion of the umbilical cord: a prospective color Doppler ultrasound study. Ultrasound in obstetrics & gynecology : the official journal of the International Society of Ultrasound in Obstetrics and Gynecology. 2003;21(6):564-9. Epub 2003/06/17. doi: 10.1002/uog.132. PubMed PMID: 12808673.

11. Catanzarite V, Maida C, Thomas W, Mendoza A, Stanco L, Piacquadio KM. Prenatal sonographic diagnosis of vasa previa: ultrasound findings and obstetric outcome in ten cases. Ultrasound in obstetrics & gynecology : the official journal of the International Society of Ultrasound in Obstetrics and Gynecology. 2001;18(2):109-15. Epub 2001/09/01. doi: 10.1046/j.1469-0705.2001.00448.x. PubMed PMID: 11529988.

12. Bronsteen R, Whitten A, Balasubramanian M, Lee W, Lorenz R, Redman M, et al. Vasa previa: clinical presentations, outcomes, and implications for management. Obstetrics and gynecology. 2013;122(2 Pt 1):352-7. Epub 2013/08/24. doi: 10.1097/AOG.0b013e31829cac58. PubMed PMID: 23969805.

13. Attilakos G, David A, Brocklehurst P, Knight M. Vasa praevia: A national UK study using the UK Obstetric Surveillance System (UKOSS). Oral Abstracts BJOG 2017;124(S2):9.

14. National Health Service. NHS Maternity Statistics, England 2016-17 2017 [29th May 2018]. Available from: <https://digital.nhs.uk/data-and-information/publications/statistical/nhs-maternity-statistics/2016-17>.

15. Jauniaux E, Alfirevic Z, Bhide A, Burton GJ, Collins S, Silver R. Vasa Praevia: Diagnosis and Management: Green‐top Guideline No. 27b. . BJOG: An International Journal of Obstetrics & Gynaecology. 2018.

16. UK National Screening Committee, Costello Medical. Adverse Outcomes of Velamentous Cord Insertion: Systematic Literature Review Report (Data on File). 2018.

17. Oyelese Y, Catanzarite V, Prefumo F, Lashley S, Schachter M, Tovbin Y, et al. Vasa previa: the impact of prenatal diagnosis on outcomes. Obstetrics and gynecology. 2004;103(5 Pt 1):937-42. Epub 2004/05/04. doi: 10.1097/01.aog.0000123245.48645.98. PubMed PMID: 15121568.
