# Supplementary material for: The impact of ultrasound-based antenatal screening strategies to detect vasa praevia in the United Kingdom: An exploratory study using decision analytic modelling methods
Source: PLoS One. 2022 Dec 20;17(12):e0279229. doi: 10.1371/journal.pone.0279229 (PMC9767376; doi:10.1371/journal.pone.0279229)
Supplement: S2 Table — (DOCX) [file pone.0279229.s003.docx]

S2 Table: Alternative inputs used in the Alternative Inputs scenario analysis

| **Input** | **Base Case Value** | **Reference** | **Alternative value** | **Reference** | **Rationale for selection of alternative value** |
| --- | --- | --- | --- | --- | --- |
| **Prevalence and incidence inputs** | | | | | |
| Incidence of VCI (general population) | 1.50% | Ebbing 2013 [[1](#_ENREF_1)] | 0.50% | Esakoff 2012 [[2](#_ENREF_2)] | Feasible literature value (based on applicability and quality of the study) identified through targeted searches |
| Incidence of VCI (IVF pregnancies) | 3.70% | Ebbing 2013 [[1](#_ENREF_1)] | 20.40% | Yanaihara 2018 [[3](#_ENREF_3)] | Feasible literature value (based on applicability and quality of the study) identified through targeted searches |
| Incidence of VCI (LLP pregnancies) | 2.80% | Suzuki 2015 [[4](#_ENREF_4)] | 5.60% | Ebbing 2013 [[1](#_ENREF_1)] | Feasible literature value (based on applicability and quality of the study) identified through targeted searches |
| Incidence of VP (general population) | 0.03% | UK NSC 2017 [[5](#_ENREF_5)] | 0.06% | Ruiter 2016 [[6](#_ENREF_6)] | Feasible literature value (based on applicability and quality of the study) identified through targeted searches |
| Incidence of VP (IVF pregnancies) | 0.34% | Schachter 2002 [[7](#_ENREF_7)] | 0.50% | Baulies 2007 [[8](#_ENREF_8)] | Feasible literature value (based on applicability and quality of the study) identified through targeted searches |
| Incidence of VP (LLP pregnancies) | 0.52% | Rosenberg 2011 [[9](#_ENREF_9)] | 1.20% | Baulies 2007 [[8](#_ENREF_8)] | Feasible literature value (based on applicability and quality of the study) identified through targeted searches |
| **Diagnostic inputs** | | | | | |
| Sensitivity of TAS for VCI | 99.20% | Sepulveda 2003 [[10](#_ENREF_10)] | 62.50% | Hasegawa 2006 [[11](#_ENREF_11)] | Feasible literature value (based on applicability and quality of the study) identified through targeted searches |
| Sensitivity of TAS for BL/S placenta | 75.00% | Cipriano 2010 [[12](#_ENREF_12)] | 65.00% | Cipriano 2010 [[12](#_ENREF_12)] | Lower range value supplied in the context of the applied base case value (due to non-availability of feasible alternative literature values) |
| Sensitivity of TAS for VP | 86.70% | Catanzarite 2001 [[13](#_ENREF_13)] | 70.00% | Kanda 2011 [[14](#_ENREF_14)] | Feasible literature value (based on applicability and quality of the study) identified through targeted searches |
| Sensitivity of TVS for VP | 96.60% | Bronsteen 2013 [[15](#_ENREF_15)] | 90.00% | Kanda 2011 [[14](#_ENREF_14)] | Feasible literature value (based on applicability and quality of the study) identified through targeted searches |

**Abbreviations:** BL/S, bilobed or succenturiate; IVF, in vitro fertilisation; LLP, low-lying placenta; TAS, transabdominal sonography; TVS, transvaginal sonography; UK NSC, United Kingdom National Screening Committee; VCI, velamentous cord insertion; VP, vasa praevia.

**References**

1. Ebbing C, Kiserud T, Johnsen SL, Albrechtsen S, Rasmussen S. Prevalence, risk factors and outcomes of velamentous and marginal cord insertions: a population-based study of 634,741 pregnancies. PloS one. 2013;8(7):e70380. Epub 2013/08/13. doi: 10.1371/journal.pone.0070380. PubMed PMID: 23936197; PubMed Central PMCID: PMCPMC3728211.

2. Esakoff TF, Cheng YW, Snowden J, Tran SH, Shaffer BL, Caughey AB. Velamentous cord insertion: Does it affect perinatal outcomes? American journal of obstetrics and gynecology. 2012;1):S21. doi: <http://dx.doi.org/10.1016/j.ajog.2011.10.058>. PubMed PMID: 70632793.

3. Yanaihara A, Hatakeyama S, Ohgi S, Motomura K, Taniguchi R, Hirano A, et al. Difference in the size of the placenta and umbilical cord between women with natural pregnancy and those with IVF pregnancy. J Assist Reprod Genet. 2018;35(3):431-4. Epub 11/14. doi: 10.1007/s10815-017-1084-2. PubMed PMID: 29134477.

4. Suzuki S, Kato M. Clinical Significance of Pregnancies Complicated by Velamentous Umbilical Cord Insertion Associated With Other Umbilical Cord/Placental Abnormalities. Journal of clinical medicine research. 2015;7(11):853-6. Epub 2015/10/23. doi: 10.14740/jocmr2310w. PubMed PMID: 26491497; PubMed Central PMCID: PMCPMC4596266.

5. UK National Screening Committee. Screening for vasa praevia in the second trimester of pregnacy - external review against programme appraisal criteria for the UK National Screening Committee (UK NSC). 2017.

6. Ruiter L, Kok N, Limpens J, Derks JB, de Graaf IM, Mol B, et al. Incidence of and risk indicators for vasa praevia: a systematic review. BJOG : an international journal of obstetrics and gynaecology. 2016;123(8):1278-87. Epub 2015/12/24. doi: 10.1111/1471-0528.13829. PubMed PMID: 26694639.

7. Schachter M, Tovbin Y, Arieli S, Friedler S, Ron-El R, Sherman D. In vitro fertilization is a risk factor for vasa previa. Fertility and sterility. 2002;78(3):642-3. Epub 2002/09/07. PubMed PMID: 12215350.

8. Baulies S, Maiz N, Munoz A, Torrents M, Echevarria M, Serra B. Prenatal ultrasound diagnosis of vasa praevia and analysis of risk factors. Prenatal diagnosis. 2007;27(7):595-9. Epub 2007/05/15. doi: 10.1002/pd.1753. PubMed PMID: 17497747.

9. Rosenberg T, Pariente G, Sergienko R, Wiznitzer A, Sheiner E. Critical analysis of risk factors and outcome of placenta previa. Archives of gynecology and obstetrics. 2011;284(1):47-51. Epub 2010/07/24. doi: 10.1007/s00404-010-1598-7. PubMed PMID: 20652281.

10. Sepulveda W, Rojas I, Robert JA, Schnapp C, Alcalde JL. Prenatal detection of velamentous insertion of the umbilical cord: a prospective color Doppler ultrasound study. Ultrasound in obstetrics & gynecology : the official journal of the International Society of Ultrasound in Obstetrics and Gynecology. 2003;21(6):564-9. Epub 2003/06/17. doi: 10.1002/uog.132. PubMed PMID: 12808673.

11. Hasegawa J, Matsuoka R, Ichizuka K, Otsuki K, Sekizawa A, Farina A, et al. Cord insertion into the lower third of the uterus in the first trimester is associated with placental and umbilical cord abnormalities. Ultrasound in Obstetrics & Gynecology. 2006;28(2):183-6. doi: 10.1002/uog.2839.

12. Cipriano LE, Barth Jr WH, Zaric GS. The cost-effectiveness of targeted or universal screening for vasa praevia at 18–20 weeks of gestation in Ontario. BJOG: An International Journal of Obstetrics & Gynaecology. 2010;117(9):1108-18. doi: 10.1111/j.1471-0528.2010.02621.x.

13. Catanzarite V, Maida C, Thomas W, Mendoza A, Stanco L, Piacquadio KM. Prenatal sonographic diagnosis of vasa previa: ultrasound findings and obstetric outcome in ten cases. Ultrasound in obstetrics & gynecology : the official journal of the International Society of Ultrasound in Obstetrics and Gynecology. 2001;18(2):109-15. Epub 2001/09/01. doi: 10.1046/j.1469-0705.2001.00448.x. PubMed PMID: 11529988.

14. Kanda E, Matsuda Y, Kamitomo M, Maeda T, Mihara K, Hatae M. Prenatal diagnosis and management of vasa previa: a 6-year review. J Obstet Gynaecol Res. 2011;37(10):1391-6. Epub 2011/05/24. doi: 10.1111/j.1447-0756.2011.01544.x. PubMed PMID: 21599804.

15. Bronsteen R, Whitten A, Balasubramanian M, Lee W, Lorenz R, Redman M, et al. Vasa previa: clinical presentations, outcomes, and implications for management. Obstetrics and gynecology. 2013;122(2 Pt 1):352-7. Epub 2013/08/24. doi: 10.1097/AOG.0b013e31829cac58. PubMed PMID: 23969805.
