# Supplementary material for: The impact of ultrasound-based antenatal screening strategies to detect vasa praevia in the United Kingdom: An exploratory study using decision analytic modelling methods
Source: PLoS One. 2022 Dec 20;17(12):e0279229. doi: 10.1371/journal.pone.0279229 (PMC9767376; doi:10.1371/journal.pone.0279229)
Supplement: S1 File — (DOCX) [file pone.0279229.s004.docx]

S1 File: SLR and MA on adverse perinatal outcomes

One of the aims of the review conducted by the UK NSC in 2017 was to evaluate whether VCI is associated with adverse perinatal outcomes, so as to understand what should be taken into consideration if a screening programme for VP were to include VCI as part of the screening algorithm.

The direction of the evidence on the association of VCI with perinatal death, low Apgar score and pre-eclampsia was unclear in the 2017 UK NSC review. Given that UK NSC evidence reviews are not intended to provide a quantitative evidence synthesis, an SLR followed by MAs were conducted to ascertain the association between VCI, (un-)diagnosed VP or normal pregnancy and perinatal outcomes; the MAs were also subsequently used to inform model inputs. Although the SLR and MAs included all three outcomes that were unclear from the 2017 UK NSC review and are presented here, low Apgar score and pre-eclampsia were not included in the model, as, following discussions with clinical experts, perinatal mortality was judged to be the key outcome of interest when considering potential screening pathways for VP.

Systematic Literature Review

An SLR was carried out to identify all evidence on the association between VCI and perinatal death, low Apgar score and pre-eclampsia. MEDLINE, Embase and the Cochrane Library database were searched from inception until 30th January 2018. Proceedings of major congresses conducted since 2016, as well as reference lists of SLRs and MAs identified during the review, were hand-searched. Records were screened at abstract and full text stages by two independent reviewers based on pre-defined eligibility criteria (Table 1). Studies were eligible for inclusion if they reported perinatal death, Apgar score or pre-eclampsia in women with pregnancies affected by VCI.

Table 1: SLR inclusion and exclusion criteria

| Domain | Target condition | Population | Outcome | Study type | Setting | Other considerations |
| --- | --- | --- | --- | --- | --- | --- |
| Inclusion criteria | VCI | Women with pregnancies affected by VCI | - Perinatal death (neonatal and/or fetal death [death of the baby occurring at any point during pregnancy or up to 28 days post-partum]) - Pre-eclampsia (developed during or after pregnancy) - Low Apgar score | - SLRs and MAs - Observational studies - Cross-sectional studies - Interventional studies including a usual care arm^a^ | UK^b^, EEA and OECD member countries, excluding South Korea and Mexico |  |
| Exclusion criteria |  | Studies that do not include pregnant women with VCI  Studies that consider pregnant women with VCI but do not present outcomes for this population separately to outcomes for irrelevant populations | Lack of eligible outcomes reported | - Intervention studies without a usual care arm^a^ - Case reports/case series - Narrative reviews, commentaries, opinion pieces, editorials | Studies in non-eligible countries or international studies that consider eligible and non-eligible countries but outcomes are not presented separately to outcomes from non-eligible countries | Studies with full text not in English language |

^a^ Usual care arm is one where women are treated the same as they would normally experience in standard clinical care. ^b^ Studies in the UK were to be prioritised for extraction. However, as it was anticipated that UK studies may be limited, studies from other countries where the pathway of care for pregnant women is similar to that in the UK were also eligible for inclusion.

**Abbreviations:** Apgar, appearance, pulse, grimace, activity and respiration; EEA, European Economic Area; MAs, meta-analyses; OECD, Organisation for Economic Co-Operation and Development; SLR, systematic literature review; VCI, velamentous cord insertion.

Studies judged relevant for inclusion in the SLR were extracted and their quality was appraised using an adapted checklist based on the JBI Critical Appraisal Checklist for Studies Reporting Prevalence Data [[1](#_ENREF_1)] and the Centre for Evidence Based Medicine Prognostic Studies Critical Appraisal Worksheet. [[2](#_ENREF_2)] This was performed by one reviewer and checked by a second, independent reviewer.

A Preferred Reporting Items for Systematic Reviews and Meta-Analyses (PRISMA) flowchart summarising the publications included and excluded at each stage of the review is presented in Fig 1. A total of 34 unique studies reported by 37 publications were included in the SLR. Most studies were of a retrospective or case-control design (Table 2), and overall at a moderate risk of bias. No studies conducted in the UK were identified.

Fig 1: PRISMA flowchart of records included and excluded at each stage of the review


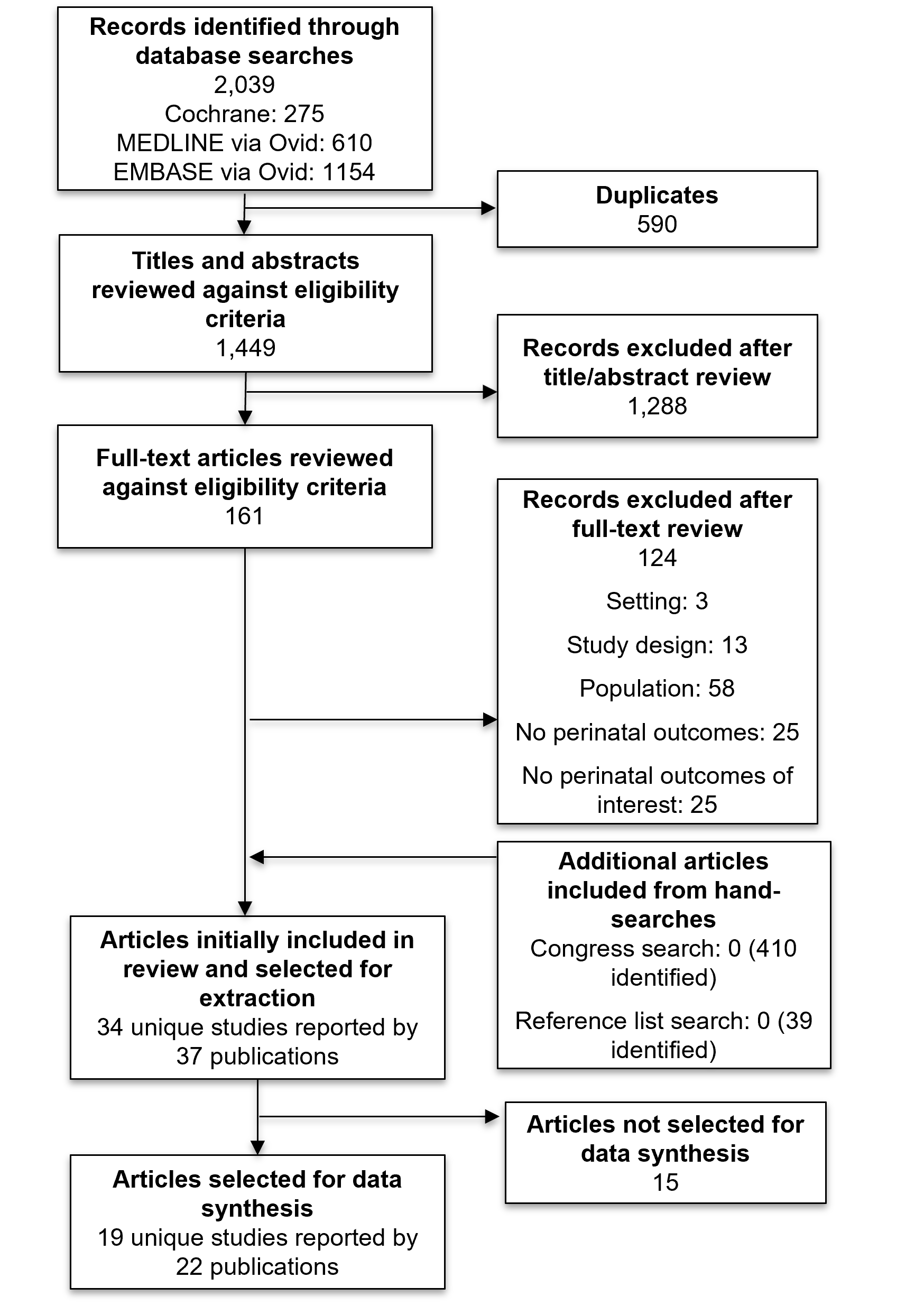


Meta Analyses

Following on from the SLR, initial MAs were conducted to estimate the increased odds of each reported outcome (perinatal death, low Apgar score, pre-eclampsia) in pregnancies with VCI compared to pregnancies without VCI. Studies were included in the MAs if they reported data in the form of number of participants per exposed (VCI) or control (non-VCI) groups; studies only reporting outcomes as odds ratios (ORs) with 95% CIs were excluded. A summary of studies considered for inclusion in the MAs, and the reason for exclusion where applicable, is provided in Table 2. Further MAs were conducted to also estimate the increased odds of emergency Caesarean section, pre-term birth, admission to NICU and small for gestational age (SGA) in pregnancies with VCI compared to pregnancies without VCI; these calculations were based on evidence identified through the 2017 UK NSC review and any additional data reported in studies identified by the SLR. [[3](#_ENREF_3)]

In order to provide relevant inputs for the inclusion of perinatal outcomes in the model, additional MAs were conducted to estimate the overall prevalence of selected outcomes in populations with VCI, without VCI, with antenatally diagnosed VP and with undiagnosed VP.

Both fixed effect and random effects logistic regression models were considered for each set of data, but random effects models were deemed more suitable due to the heterogeneous nature of the studies and were used where possible. In cases where the random effects model failed to converge due to the limited data available, fixed effects models were used (which all achieved convergence). For the calculation of ORs, sensitivity analyses including only studies where VCI was diagnosed routinely at birth (see Table 2 for a summary of which studies were included) were also conducted to minimise the risk of ascertainment bias. All of these were consistent with the base case results in that the association of VCI with the perinatal outcome examined remained statistically significant.

The results of all MAs conducted are summarised in Table 3 (for the increased odds of adverse perinatal outcomes in VCI versus non-VCI pregnancies) and Table 4 (for the overall prevalence of adverse perinatal outcomes by pregnancy type), with selected results presented in Table 4 also serving as corresponding model inputs. With regards to the odds ratios presented in Table 3, although the MAs indicate that there is a statistically significant association between VCI and all examined perinatal outcomes, the association is weak (except perinatal death, where it is moderate); as such, its clinical relevance remains unclear.

Table 2: Studies considered for inclusion in the initial MA

| Study | Study design | Population | Reason(s) for exclusion from MAs^a^ | Inclusion in sensitivity analyses |
| --- | --- | --- | --- | --- |
| Baumfeld 2016 [[4](#_ENREF_4)] | Retrospective case-control | Singleton and multiple pregnancies | N/A | **N** |
| Bjoro Jr 1983 [[5](#_ENREF_5)] | Prospective cohort | NR | Data for only one study group was reported (no control group) | N/A |
| Boulis 2013 [[6](#_ENREF_6)] | Retrospective chart review | Singleton pregnancies | Data for only one study group was reported (no control group) | N/A |
| Bukowski 2017 [[7](#_ENREF_7)] | Case-control | Singleton pregnancies | Insufficient outcome data (n/N was not reported) | N/A |
| Chan 2012 [[8](#_ENREF_8)] | Case-control | Singleton pregnancies | Insufficient outcome data (n/N not reported for perinatal death, p-value only)  Apgar score reported as a mean value | N/A |
| Cirstoiu 2016 [[9](#_ENREF_9)] | Retrospective | NR | Data for only one study group was reported (no control group) | N/A |
| Costa-Castro 2013 [[10](#_ENREF_10), [11](#_ENREF_11)] | Retrospective cohort | Twin pregnancies | N/A | **N** |
| Costa-Castro 2016 [[12](#_ENREF_12)] | Retrospective cohort | Twin pregnancies | Insufficient outcome data (n/N not reported) | N/A |
| Ebbing 2013 [[13](#_ENREF_13)], Ebbing 2015 [[14](#_ENREF_14)] | Retrospective cohort | Singleton and twin pregnancies^b^ | N/A | **Y** |
| Eddleman 1992 [[15](#_ENREF_15)] | Retrospective cohort | Singleton pregnancies | N/A | **Y** |
| Esakoff 2012 [[16](#_ENREF_16)] | Retrospective cohort | Singleton pregnancies | Insufficient outcome data (n/N was not reported) | N/A |
| Esakoff 2015 [[17](#_ENREF_17)] | Retrospective cohort | Singleton pregnancies | N/A | **N** |
| Feldman 2002 [[18](#_ENREF_18)] | Retrospective cohort | Triplet pregnancies | N/A | **N** |
| Fries 1993 [[19](#_ENREF_19)] | Retrospective cohort | Twin pregnancies | Unclear how data was collected and how the outcome was calculated | – |
| Hack 2008 [[20](#_ENREF_20)] | Prospective cohort | Twin pregnancies | N/A | **N** |
| Hack 2009 [[21](#_ENREF_21)] | Prospective cohort | Twin pregnancies | N/A | **N** |
| Hasegawa 2009, Hasegawa 2009 [[22](#_ENREF_22), [23](#_ENREF_23)] | Retrospective cohort | Singleton pregnancies | NA | **N** |
| Hasegawa 2011 [[24](#_ENREF_24)] | Case-control | Singleton pregnancies | N/A | **N** |
| Heinonen 1996 [[25](#_ENREF_25)] | Retrospective cohort | Singleton pregnancies | N/A | **Y** |
| Ismail 2017 [[26](#_ENREF_26)] | Prospective cohort | Singleton pregnancies | N/A | **N** |
| Lauslahti 1979 [[27](#_ENREF_27)] | Cohort | NR | N/A | **N** |
| Liu 1992 [[28](#_ENREF_28)] | Retrospective cohort | Multiple pregnancies | Cohort overlaps with Robinson 1983 (which is of higher quality than Liu 1992) | – |
| Nakamura 2017 [[29](#_ENREF_29)] | Retrospective cohort | Singleton and multiple pregnancies | Apgar score reported as a median value (1 min, 5 min) | – |
| Paavonen 1984 [[30](#_ENREF_30)] | Retrospective cohort | Singleton pregnancies | Data for only one study group was reported (no control group) | – |
| Pinar 2012 [[31](#_ENREF_31)] | Case-control | Singleton pregnancies | n/N not reported | – |
| Raisanen 2012 [[32](#_ENREF_32)] | Retrospective cohort | Singleton pregnancies | N/A | **Y** |
| Rand 2009 [[33](#_ENREF_33)] | Retrospective cohort | Twin pregnancies | Insufficient outcome data (n/N not reported and data reported for one study group) | – |
| Robinson 1983 [[34](#_ENREF_34)] | Retrospective cohort | Singleton and twin pregnancies | N/A | **Y** |
| Simpson 2011 [[35](#_ENREF_35)] | Retrospective cohort | Twin pregnancies | N/A | **N** |
| Sinkin 2018 [[36](#_ENREF_36)] | Retrospective case-control | Singleton and twin pregnancies | N/A | **N** |
| Suzuki 2015 [[37](#_ENREF_37)] | Retrospective | Singleton pregnancies | N/A | **Y** |
| Takita 2018 [[38](#_ENREF_38)] | Retrospective cohort | Singleton and multiple pregnancies | Data for only one study group was reported (no control group) | – |
| Waszak 2016 [[39](#_ENREF_39)] | Prospective cohort | Twin pregnancies | Data for only one study group was reported (no control group) and Apgar score reported as a mean value | – |
| Yerlikaya 2016 [[40](#_ENREF_40)] | Retrospective case-control | Singleton pregnancies | Apgar score reported as a mean value | **N** |

^a^ For studies marked as N/A (not applicable) all relevant outcomes reported in the study have been included in the MA. b Only data for singletons from Ebbing 2013 was used in the analysis.

**Abbreviations:** Apgar, appearance, pulse, grimace, activity and respiration; MA, meta-analyses; N, No; N/A, not applicable; NR, not reported; Y, yes.

Table 3: Odds of adverse perinatal outcomes in VCI (relative to non-VCI pregnancies)

| Outcome | OR (95% CI) | Sensitivity Analysis OR (95% CI) |
| --- | --- | --- |
| Perinatal death | 3.30 (2.29–4.76) | 2.17 (1.86–2.54) |
| Low Apgar Score at 1 minute | 1.76 (1.43–2.17) | 1.62 (1.28–2.04) |
| Low Apgar Score at 5 minutes | 1.97 (1.77–2.20) | 1.96 (1.75–2.19) |
| Pre-term birth | 2.22 (1.80–2.75) | 2.08 (1.95–2.22) |
| Emergency Caesarean section ^a^ | 2.01 (1.66–2.42) | N/A ^b^ |
| Admission to NICU | 1.90 (1.80–2.01) | 1.92 (1.81–2.03) |
| Pre-eclampsia | 1.45 (1.25–1.69) | 1.58 (1.45–1.72) |
| Small for gestational age | 1.86 (1.76–1.96) | 1.88 (1.77–2.00) |

^a^ Studies where delivery was only defined as “Caesarean section” as opposed to specifying an emergency Caesarean section were not included in this analysis. ^b^ A sensitivity analysis was not performed as VCI was routinely diagnosed at birth in all studies.

**Abbreviations**: Apgar, appearance, pulse, grimace, activity and respiration; CI, confidence interval; N/A, not applicable; NICU, neonatal intensive care unit; OR, odds ratio; VCI, velamentous cord insertion.

Table 4: Prevalence of selected adverse perinatal outcomes in VCI, diagnosed and undiagnosed VP, and normal (control) pregnancies

| Outcome | Prevalence in VCI (95% CI) | Prevalence in controls ^a^ (95% CI) | Prevalence in prenatally diagnosed VP (95% CI) | Prevalence in VP not diagnosed prenatally (95% CI) |
| --- | --- | --- | --- | --- |
| Emergency Caesarean section ^b^ | 0.14 (0.14–0.15) | 0.07 (0.05–0.11) | 0.23 (0.13–0.36) | 0.68 (0.59–0.76) |
| Perinatal death | 0.02 (0.01–0.04) | 0.01 (0.00–0.01) | 0.03 (0.01–0.07) | 0.36 (0.09–0.75) |
| Admission to NICU | 0.15 (0.09–0.24) | 0.11 (0.06-0.20) | 0.22 (0.02–0.78) | N/A^c^ |
| Low Apgar Score at 1 minute | 0.11 (0.07–0.18) | 0.06 (0.04–0.10) | 0.20 (0.09–0.38) | 0.70 (0.38–0.90) |
| Low Apgar Score at 5 minutes | 0.04 (0.03–0.06) | 0.02 (0.01–0.02) | N/A ^d^ | N/A ^d^ |
| Pre-term birth | 0.15 (0.10–0.21) | 0.07 (0.05–0.08) | 0.89 (0.49–0.99) | N/A^c^ |

^a^ Defined as non-VCI pregnancies. ^b^ Studies where delivery was only defined as “Caesarean section” as opposed to specifying an emergency Caesarean section were not included in this analysis. ^c^ No studies reporting admission to NICU or pre-term birth in undiagnosed VP were identified ^d^A single study reporting the risk of low Apgar score at 5 minutes was identified in the 2017 review. As such, no MA was conducted and a more recently published estimate was used in the model (Baumfeld 2016).

**Abbreviations:** Apgar, appearance, pulse, grimace, activity and respiration; CI, confidence interval; N/A, not applicable; NICU, neonatal intensive care unit; VCI, velamentous cord insertion; VP, vasa praevia.

**References**

1. Munn Z, Moola S, Riitano D, Lisy K. The development of a critical appraisal tool for use in systematic reviews addressing questions of prevalence. Int J Health Policy Manag. 2014;3(3):123-8. doi: 10.15171/ijhpm.2014.71. PubMed PMID: 25197676.

2. Centre for Evidence-Based Medicine. Critical Appraisal of Prognostic Studies 2018. Available from: <http://www.Cebm.Net/Critical-Appraisal/>.

3. UK National Screening Committee. Screening for vasa praevia in the second trimester of pregnacy - external review against programme appraisal criteria for the UK National Screening Committee (UK NSC). 2017.

4. Baumfeld Y, Gutvirtz G, Shoham I, Sheiner E. Fetal heart rate patterns of pregnancies with vasa previa and velamentous cord insertion. Archives of gynecology and obstetrics. 2016;293(2):361-7.

5. Bjoro Jr K. Vascular anomalies of the umbilical cord. II. Perinatal and pediatric implications. Early Human Development. 1983;8(3-4):279-87. PubMed PMID: 14232940.

6. Boulis TS, Rochelson B, Meirowitz N, Fleischer A, Smith-Levitin M, Edelman M, et al. Are there adverse pregnancy outcomes associated with concordant or discordant velamentous/marginal cord insertion in twins? American journal of obstetrics and gynecology. 2013;208 (1 SUPPL.1):S78-S9. doi: <http://dx.doi.org/10.1016/j.ajog.2012.10.324>. PubMed PMID: 70967177.

7. Bukowski R, Hansen NI, Pinar H, Willinger M, Reddy UM, Parker CB, et al. Altered fetal growth, placental abnormalities, and stillbirth. PLoS ONE [Electronic Resource]. 2017;12(8):e0182874. doi: <https://dx.doi.org/10.1371/journal.pone.0182874>. PubMed PMID: 28820889.

8. Chan JS, Baergen RN. Gross umbilical cord complications are associated with placental lesions of circulatory stasis and fetal hypoxia. Pediatr Dev Pathol. 2012;15(6):487-94. doi: <https://dx.doi.org/10.2350/12-06-1211-OA.1>. PubMed PMID: 22978619.

9. Cirstoiu MM, Turcan N, BrAtil AE, Munteanu O, Bodean O, Voicu D, et al. Velamentous cord insertion - An important obstetrical risk factor. Ginecoeu. 2016;12(3):129-34. doi: <http://dx.doi.org/10.18643/gieu.2016.129>. PubMed PMID: 612944692.

10. Costa-Castro T, De Villiers S, Montenegro N, Severo M, Oepkes D, Matias A, et al. Velamentous cord insertion in monochorionic twins: Does it matter? Prenatal diagnosis. 2013;1):13. doi: <http://dx.doi.org/10.1002/pd.4147>. PubMed PMID: 71166399.

11. Costa-Castro T, De Villiers S, Montenegro N, Severo M, Oepkes D, Matias A, et al. Velamentous cord insertion in monochorionic twins with or without twin-twin transfusion syndrome: Does it matter? Placenta. 2013;34(11):1053-8. doi: <http://dx.doi.org/10.1016/j.placenta.2013.08.009>. PubMed PMID: 370132576.

12. Costa-Castro T, Zhao DP, Lipa M, Haak MC, Oepkes D, Severo M, et al. Velamentous cord insertion in dichorionic and monochorionic twin pregnancies - Does it make a difference? Placenta. 2016;42:87-92. doi: <http://dx.doi.org/10.1016/j.placenta.2016.04.007>. PubMed PMID: 609847393.

13. Ebbing C, Kiserud T, Johnsen SL, Albrechtsen S, Rasmussen S. Prevalence, risk factors and outcomes of velamentous and marginal cord insertions: a population-based study of 634,741 pregnancies. PloS one. 2013;8(7):e70380. Epub 2013/08/13. doi: 10.1371/journal.pone.0070380. PubMed PMID: 23936197; PubMed Central PMCID: PMCPMC3728211.

14. Ebbing C, Kiserud T, Johnsen SL, Albrechtsen S, Rasmussen S. Third stage of labor risks in velamentous and marginal cord insertion: a population‐based study. Acta Obstet Gynecol Scand. 2015;94(8):878-83.

15. Eddleman KA, Lockwood CJ, Berkowitz GS, Lapinski RH, Berkowitz RL. Clinical significance and sonographic diagnosis of velamentous umbilical cord insertion. American Journal of Perinatology. 1992;9(2):123-6. PubMed PMID: 22127694.

16. Esakoff TF, Cheng YW, Snowden J, Tran SH, Shaffer BL, Caughey AB. Velamentous cord insertion: Does it affect perinatal outcomes? American journal of obstetrics and gynecology. 2012;1):S21. doi: <http://dx.doi.org/10.1016/j.ajog.2011.10.058>. PubMed PMID: 70632793.

17. Esakoff TF, Cheng YW, Snowden JM, Tran SH, Shaffer BL, Caughey AB. Velamentous cord insertion: Is it associated with adverse perinatal outcomes? Journal of Maternal-Fetal and Neonatal Medicine. 2015;28(4):409-12. doi: <http://dx.doi.org/10.3109/14767058.2014.918098>. PubMed PMID: 602903819.

18. Feldman DM, Borgida AF, Trymbulak WP, Barsoom MJ, Sanders MM, Rodis JF. Clinical implications of velamentous cord insertion in triplet gestations. American journal of obstetrics and gynecology. 2002;186(4):809-11. doi: <http://dx.doi.org/10.1067/mob.2002.121653>. PubMed PMID: 34494368.

19. Fries MH, Goldstein RB, Kilpatrick SJ, Golbus MS, Callen PW, Filly RA. The role of velamentous cord insertion in the etiology of twin-twin transfusion syndrome. Obstetrics and gynecology. 1993;81(4):569-74. PubMed PMID: 23122666.

20. Hack KE, Nikkels PG, Koopman-Esseboom C, Derks JB, Elias SG, van Gemert MJ, et al. Placental characteristics of monochorionic diamniotic twin pregnancies in relation to perinatal outcome. Placenta. 2008;29(11):976-81. doi: <https://dx.doi.org/10.1016/j.placenta.2008.08.019>. PubMed PMID: 18835495.

21. Hack KE, van Gemert MJ, Lopriore E, Schaap AH, Eggink AJ, Elias SG, et al. Placental characteristics of monoamniotic twin pregnancies in relation to perinatal outcome. Placenta. 2009;30(1):62-5. doi: <https://dx.doi.org/10.1016/j.placenta.2008.09.016>. PubMed PMID: 19010539.

22. Hasegawa J, Matsuoka R, Ichizuka K, Kotani M, Nakamura M, Mikoshiba T, et al. Atypical variable deceleration in the first stage of labor is a characteristic fetal heart-rate pattern for velamentous cord insertion and hypercoiled cord. Journal of Obstetrics and Gynaecology Research. 2009;35(1):35-9. doi: <http://dx.doi.org/10.1111/j.1447-0756.2008.00863.x>. PubMed PMID: 354117481.

23. Hasegawa J, Matsuoka R, Ichizuka K, Nakamura M, Sekizawa A, Okai T. Do fetal heart rate deceleration patterns during labor differ between various umbilical cord abnormalities? Journal of Perinatal Medicine. 2009;37(3):276-80. doi: <http://dx.doi.org/10.1515/JPM.2009.039>. PubMed PMID: 354430761.

24. Hasegawa J, Sekizawa A, Farina A, Nakamura M, Matsuoka R, Ichizuka K, et al. Location of the placenta or the umbilical cord insertion site in the lowest uterine segment is associated with low maternal blood pressure. BJOG: An International Journal of Obstetrics and Gynaecology. 2011;118(12):1464-9. doi: <http://dx.doi.org/10.1111/j.1471-0528.2011.03051.x>. PubMed PMID: 51523910.

25. Heinonen S, Ryynanen M, Kirkinen P, Saarikoski S. Velamentous umbilical cord insertion may be suspected from maternal serum alpha-fetoprotein and hCG. Br J Obstet Gynaecol. 1996;103(3):209-13. PubMed PMID: 8630303.

26. Ismail KI, Hannigan A, Fitzgerald B, Kelehan P, O'Donoghue K, Cotter A. Placental and umbilical cord morphometry of pregnancies with small-for-gestational-age infants. BJOG: An International Journal of Obstetrics and Gynaecology. 2017;124 (Supplement 2):27. doi: <http://dx.doi.org/10.1111/1471-0528.14586>. PubMed PMID: 615222944.

27. Lauslahti K, Ikonen S. Placenta as an indicator of fetal postnatal prognosis. Acta Obstet Gynecol Scand. 1979;58(2):163-7. PubMed PMID: 9166623.

28. Liu S, Benirschke K, Scioscia AL, Mannino FL. Intrauterine death in multiple gestation. Acta Geneticae Medicae et Gemellologiae. 1992;41(1):5-26. PubMed PMID: 22342640.

29. Nakamura M, Umehara N, Ishii K, Sasahara J, Kiyoshi K, Ozawa K, et al. A poor long-term neurological prognosis is associated with abnormal cord insertion in severe growth-restricted fetuses. Journal of Perinatal Medicine. 2017;21. doi: <http://dx.doi.org/10.1515/jpm-2017-0240>. PubMed PMID: 620194280.

30. Paavonen J, Jouttunpaa K, Kangasluoma P. Velamentous insertion of the umbilical cord and vasa previa. International Journal of Gynecology and Obstetrics. 1984;22(3):207-11. PubMed PMID: 14119984.

31. Pinar H, Dudley D. Histologic features of the placenta in stillbirth: Results of a case control study. American journal of obstetrics and gynecology. 2012;1):S61. doi: <http://dx.doi.org/10.1016/j.ajog.2011.10.124>. PubMed PMID: 70632859.

32. Raisanen S, Georgiadis L, Harju M, Keski-Nisula L, Heinonen S. Risk factors and adverse pregnancy outcomes among births affected by velamentous umbilical cord insertion: A retrospective population-based register study. European Journal of Obstetrics Gynecology and Reproductive Biology. 2012;165(2):231-4. doi: <http://dx.doi.org/10.1016/j.ejogrb.2012.08.021>. PubMed PMID: 52187958.

33. Rand L, Smith-Bindman R, Saadai P, Machin G, Feldstein V. Placental predictors of adverse outcomes in monochorionic twins. American journal of obstetrics and gynecology. 2009;1):S67. doi: <http://dx.doi.org/10.1016/j.ajog.2009.10.157>. PubMed PMID: 70128706.

34. Robinson LK, Jones KL, Benirschke K. The nature of structural defects associated with velamentous and marginal insertion of the umbilical cord. American journal of obstetrics and gynecology. 1983;146(2):191-3. PubMed PMID: 13092767.

35. Simpson LL, Vink J, Montero F, Sela HY, D'Alton M, Fuchs K, et al. Abnormal placental cord insertion in monochorionicdiamniotic twins: An ominous finding. American journal of obstetrics and gynecology. 2011;204 (1 SUPPL.):S145-S6. doi: <http://dx.doi.org/10.1016/j.ajog.2010.10.376>. PubMed PMID: 70328370.

36. Sinkin JA, Craig WY, Jones M, Pinette MG, Wax JR. Perinatal Outcomes Associated With Isolated Velamentous Cord Insertion in Singleton and Twin Pregnancies. Journal of Ultrasound in Medicine. 2018;37(2):471-8. doi: <https://dx.doi.org/10.1002/jum.14357>. PubMed PMID: 28850682.

37. Suzuki S, Kato M. Clinical Significance of Pregnancies Complicated by Velamentous Umbilical Cord Insertion Associated With Other Umbilical Cord/Placental Abnormalities. Journal of clinical medicine research. 2015;7(11):853-6. Epub 2015/10/23. doi: 10.14740/jocmr2310w. PubMed PMID: 26491497; PubMed Central PMCID: PMCPMC4596266.

38. Takita H, Hasegawa J, Nakamura M, Arakaki T, Oba T, Matsuoka R, et al. Causes of intrauterine fetal death are changing in recent years. Journal of Perinatal Medicine. 2018;46(1):97-101. doi: <https://dx.doi.org/10.1515/jpm-2016-0337>. PubMed PMID: 28236631.

39. Waszak M, Cieslik K, Pietryga M, Lewandowski J, Chuchracki M, Nowak-Markwitz E, et al. Effect of morphological and functional changes in the secundines on biometric parameters of newborns from dichorionic twin pregnancies. Ginekol Pol. 2016;87(11):755-62. doi: <https://dx.doi.org/10.5603/GP.2016.0083>. PubMed PMID: 27958634.

40. Yerlikaya G, Pils S, Springer S, Chalubinski K, Ott J. Velamentous cord insertion as a risk factor for obstetric outcome: a retrospective case-control study. Archives of gynecology and obstetrics. 2016;293(5):975-81. doi: <http://dx.doi.org/10.1007/s00404-015-3912-x>. PubMed PMID: 606613477.
