# Supplementary material for: The impact of ultrasound-based antenatal screening strategies to detect vasa praevia in the United Kingdom: An exploratory study using decision analytic modelling methods
Source: PLoS One. 2022 Dec 20;17(12):e0279229. doi: 10.1371/journal.pone.0279229 (PMC9767376; doi:10.1371/journal.pone.0279229)
Supplement: S2 File — (DOCX) [file pone.0279229.s005.docx]

S2 File: Quality assessment results

Summaries of the quality assessment result of all published sources included in the base case model are presented below.

Quality assessments of epidemiological and prognostic studies were based on the Joanna Briggs Institute Critical Appraisal Checklist for Studies Reporting Prevalence Data and the Centre for Evidence Based Medicine Prognostic Studies Critical Appraisal Worksheet (Table 1). [[1](#_ENREF_1), [2](#_ENREF_2)]

Quality assessments of diagnostics studies were based on the QUADAS-2 tool (Table 2). [[3](#_ENREF_3)]

Quality assessments of economic evaluations were based on the Drummond checklist (Table 3). [[4](#_ENREF_4)]

Table 1: Quality assessment of epidemiological and prognostic studies

|  | Baumfeld 2016 [[5](#_ENREF_5)] | Ebbing 2013 [[6](#_ENREF_6)] | Bronsteen 2013 [[7](#_ENREF_7)] | Manikanta Reddy 2013 [[8](#_ENREF_8)] | Oyelese 2004 [[9](#_ENREF_9)] | Romundstad 2006 [[10](#_ENREF_10)] | Rosenberg 2011 [[11](#_ENREF_11)] | Schachter 2002 [[12](#_ENREF_12)] | Suzuki 2015 [[13](#_ENREF_13)] |
| --- | --- | --- | --- | --- | --- | --- | --- | --- | --- |
| **Patient selection** | | | | | | | | | |
| Was the sample representative of the target population? | U | Y | Y | U | Y | Y | N | Y | N |
| Were study participants recruited in an appropriate way? | Y | Y | Y | Y | Y | Y | Y | Y | Y |
| Was the sample size adequate? | NA | Y | Y | Y | Y | Y | U | Y | Y |
| Were the study subjects and the setting described in detail? | N | Y | N | N | Y | Y | Y | N | Y |
| **Study analysis** | | | | | | | | | |
| Was the data analysis conducted with sufficient coverage of the identified sample? | Y | Y | Y | Y | Y | Y | Y | Y | Y |
| Were objective, standard criteria used for the measurement of the condition? | U | Y | U | U | Y | U | U | U | U |
| Was the condition measured reliably? | U | U | Y | U | Y | Y | Y | U | U |
| Was there appropriate statistical analysis? | Y | Y | Y | U | Y | Y | NA | Y | Y |
| Are all important confounding factors/ subgroups/differences identified and accounted for? | U | N | N | U | NA | Y | N | N | N |
| Were subpopulations identified using objective criteria? | NA | U | NA | NA | Y | Y | Y | Y | U |
| **QA of prognostic studies** | | | | | | | | | |
| Was patient follow-up sufficiently long and complete? | Y | Y | Y | U | Y | Y | Y | Y | Y |
| Were outcome criteria either objective or applied in a ‘blind’ fashion? | U | U | Y | Y | Y | Y | NA | Y | NA |
| If subgroups with different prognoses are identified, did adjustment for important prognostic factors take place? | N | Y | N | NA | Y | Y | NA | N | NA |

**Abbreviations:** N, no; NA, not applicable; QA, quality assessment; U, unclear; Y, yes.

Table 2: Quality assessment of diagnostic studies

|  | Bronsteen 2013 [[7](#_ENREF_7)] | Catanzarite 2001 [[14](#_ENREF_14)] | Sepulveda 2003 [[15](#_ENREF_15)] |
| --- | --- | --- | --- |
| **Patient selection** | | | |
| Was a consecutive or random sample of patients enrolled? | Y | Y | Y |
| Was a case-control design avoided? | Y | Y | Y |
| Did the study avoid inappropriate exclusions? | Y | Y | N |
| Could the selection of patients have introduced bias? (Risk: Low, Unclear, High) | High | Low | High |
| **Index tests** | | | |
| Were the index test results interpreted without knowledge of the reference standard? | Y | Y | Y |
| If a threshold was used, was it pre-specified? | Y | Y | Y |
| Could the conduct or interpretation of the index test have introduced bias? (Risk: Low, Unclear, High) | High | High | High |
| Is there concern that the index test, its conduct, or interpretation differ from the review question? (Concern: Low, Unclear, High) | High | High | High |
| **Reference standard** | | | |
| Is the reference standard likely to correctly classify the test condition? | U | U | Y |
| Were the reference standard results interpreted without knowledge of the results of the index test? | N | N | U |
| Could the reference standard, its conduct, or its interpretation have introduced bias? (Risk: Low, Unclear, High) | High | High | Unclear |
| Is there concern that the target condition as defined by the reference standard does not match the review question? (Concern: Low, Unclear, High) | Low | Low | Low |
| **Patient flow** | | | |
| Was there an appropriate interval between the index test(s) and the reference standard? | N | N | N |
| Did all patients receive a reference standard? | N | U | Y |
| Did patients receive the same reference standard? | Y | U | Y |
| Were all patients included in the analysis? | Y | Y | Y |
| Could the patient flow have introduced bias? (Risk: Low, Unclear, High) | High | High | High |

**Abbreviations**: N, no; U, unclear; Y, yes.

Table 3: Quality assessment of economic evaluations

|  | Cipriano 2010 [[16](#_ENREF_16)] |
| --- | --- |
| **Study design** | |
| Was the research question stated? | Y |
| Was the economic importance of the research question stated? | Y |
| Was/were the viewpoint(s) of the analysis clearly stated and justified? | Y |
| Was a rationale reported for the choice of the alternative programs or interventions compared? | Y |
| Were the alternatives being compared clearly described? | Y |
| Was the form of economic evaluation stated? | Y |
| Was the choice of form of economic evaluation justified in relation to the questions addressed? | U |
| **Data collection** | |
| Was/were the source(s) of effectiveness estimates used stated? | Y |
| Were details of the design and results of the effectiveness study given (if based on a single study)? | NA |
| Were details of the methods of synthesis or meta-analysis of estimates given (if based on an overview of a number of effectiveness studies)? | NA |
| Were the primary outcome measure(s) for the economic evaluation clearly stated? | Y |
| Were the methods used to value health states and other benefits stated? | Y |
| Were the details of the subjects from whom valuations were obtained given? | NA |
| Were productivity changes (if included) reported separately? | Y |
| Was the relevance of productivity changes to the study question discussed? | N |
| Were quantities of resources reported separately from their unit cost? | Y |
| Were the methods for the estimation of quantities and unit costs described? | Y |
| Were currency and price data recorded? | Y |
| Were details of price adjustments for inflation or currency conversion given? | Y |
| Were details of any model used given? | Y |
| Was there a justification for the choice of model used and the key parameters on which it was based? | Y |
| **Analysis and interpretation of results** | |
| Was the time horizon of cost and benefits stated? | Y |
| Was the discount rate stated? | Y |
| Was the choice of rate justified? | N |
| Was an explanation given if cost or benefits were not discounted? | NA |
| Were the details of statistical test(s) and confidence intervals given for stochastic data? | Y |
| Was the approach to sensitivity analysis described? | Y |
| Was the choice of variables for sensitivity analysis justified? | N |
| Were the ranges over which the parameters were varied stated? | Y |
| Were relevant alternatives compared in the incremental analysis? | Y |
| Was an incremental analysis reported? | Y |
| Were major outcomes presented in a disaggregated as well as aggregated form? | Y |
| Was the answer to the study question given? | Y |
| Did conclusions follow from the data reported? | Y |
| Were conclusions accompanied by the appropriate caveats? | Y |

**Abbreviations**: N, no; NA, not applicable; U, unclear; Y, yes.

**References**

1. Centre for Evidence-Based Medicine. Critical Appraisal of Prognostic Studies 2018. Available from: <http://www.Cebm.Net/Critical-Appraisal/>.

2. Munn Z, Moola S, Riitano D, Lisy K. The development of a critical appraisal tool for use in systematic reviews addressing questions of prevalence. Int J Health Policy Manag. 2014;3(3):123-8. doi: 10.15171/ijhpm.2014.71. PubMed PMID: 25197676.

3. Whiting PF, Rutjes AW, Westwood ME, Mallett S, Deeks JJ, Reitsma JB, et al. QUADAS-2: a revised tool for the quality assessment of diagnostic accuracy studies. Annals of internal medicine. 2011;155(8):529-36. Epub 2011/10/19. doi: 10.7326/0003-4819-155-8-201110180-00009. PubMed PMID: 22007046.

4. Drummond MF, Jefferson TO. Guidelines for authors and peer reviewers of economic submissions to the BMJ. The BMJ Economic Evaluation Working Party. BMJ (Clinical research ed). 1996;313(7052):275-83. Epub 1996/08/03. doi: 10.1136/bmj.313.7052.275. PubMed PMID: 8704542; PubMed Central PMCID: PMCPMC2351717.

5. Baumfeld Y, Gutvirtz G, Shoham I, Sheiner E. Fetal heart rate patterns of pregnancies with vasa previa and velamentous cord insertion. Archives of gynecology and obstetrics. 2016;293(2):361-7.

6. Ebbing C, Kiserud T, Johnsen SL, Albrechtsen S, Rasmussen S. Prevalence, risk factors and outcomes of velamentous and marginal cord insertions: a population-based study of 634,741 pregnancies. PloS one. 2013;8(7):e70380. Epub 2013/08/13. doi: 10.1371/journal.pone.0070380. PubMed PMID: 23936197; PubMed Central PMCID: PMCPMC3728211.

7. Bronsteen R, Whitten A, Balasubramanian M, Lee W, Lorenz R, Redman M, et al. Vasa previa: clinical presentations, outcomes, and implications for management. Obstetrics and gynecology. 2013;122(2 Pt 1):352-7. Epub 2013/08/24. doi: 10.1097/AOG.0b013e31829cac58. PubMed PMID: 23969805.

8. Manikanta Reddy. V, Senthil Kumar. S, Sanjeeva Reddy. N. Prevalence and Pattern of Abnormalities Occurring in Placenta and Umbilical Cord. International Journal of Medical Research and Health Sciences. 2013;2(4):935-40.

9. Oyelese Y, Catanzarite V, Prefumo F, Lashley S, Schachter M, Tovbin Y, et al. Vasa previa: the impact of prenatal diagnosis on outcomes. Obstetrics and gynecology. 2004;103(5 Pt 1):937-42. Epub 2004/05/04. doi: 10.1097/01.aog.0000123245.48645.98. PubMed PMID: 15121568.

10. Romundstad LB, Romundstad PR, Sunde A, von During V, Skjaerven R, Vatten LJ. Increased risk of placenta previa in pregnancies following IVF/ICSI; a comparison of ART and non-ART pregnancies in the same mother. Human reproduction (Oxford, England). 2006;21(9):2353-8. Epub 2006/05/27. doi: 10.1093/humrep/del153. PubMed PMID: 16728419.

11. Rosenberg T, Pariente G, Sergienko R, Wiznitzer A, Sheiner E. Critical analysis of risk factors and outcome of placenta previa. Archives of gynecology and obstetrics. 2011;284(1):47-51. Epub 2010/07/24. doi: 10.1007/s00404-010-1598-7. PubMed PMID: 20652281.

12. Schachter M, Tovbin Y, Arieli S, Friedler S, Ron-El R, Sherman D. In vitro fertilization is a risk factor for vasa previa. Fertility and sterility. 2002;78(3):642-3. Epub 2002/09/07. PubMed PMID: 12215350.

13. Suzuki S, Kato M. Clinical Significance of Pregnancies Complicated by Velamentous Umbilical Cord Insertion Associated With Other Umbilical Cord/Placental Abnormalities. Journal of clinical medicine research. 2015;7(11):853-6. Epub 2015/10/23. doi: 10.14740/jocmr2310w. PubMed PMID: 26491497; PubMed Central PMCID: PMCPMC4596266.

14. Catanzarite V, Maida C, Thomas W, Mendoza A, Stanco L, Piacquadio KM. Prenatal sonographic diagnosis of vasa previa: ultrasound findings and obstetric outcome in ten cases. Ultrasound in obstetrics & gynecology : the official journal of the International Society of Ultrasound in Obstetrics and Gynecology. 2001;18(2):109-15. Epub 2001/09/01. doi: 10.1046/j.1469-0705.2001.00448.x. PubMed PMID: 11529988.

15. Sepulveda W, Rojas I, Robert JA, Schnapp C, Alcalde JL. Prenatal detection of velamentous insertion of the umbilical cord: a prospective color Doppler ultrasound study. Ultrasound in obstetrics & gynecology : the official journal of the International Society of Ultrasound in Obstetrics and Gynecology. 2003;21(6):564-9. Epub 2003/06/17. doi: 10.1002/uog.132. PubMed PMID: 12808673.

16. Cipriano LE, Barth Jr WH, Zaric GS. The cost-effectiveness of targeted or universal screening for vasa praevia at 18–20 weeks of gestation in Ontario. BJOG: An International Journal of Obstetrics & Gynaecology. 2010;117(9):1108-18. doi: 10.1111/j.1471-0528.2010.02621.x.
