# Supplementary material for: The impact of ultrasound-based antenatal screening strategies to detect vasa praevia in the United Kingdom: An exploratory study using decision analytic modelling methods
Source: PLoS One. 2022 Dec 20;17(12):e0279229. doi: 10.1371/journal.pone.0279229 (PMC9767376; doi:10.1371/journal.pone.0279229)
Supplement: S3 File — (DOCX) [file pone.0279229.s006.docx]

S3 File: Glossary

Key terms relating to epidemiology and screening are defined below:

**Accuracy** – the overall proportion of individuals with a correct diagnosis, whether positive or negative, out of the total study cohort

**False negative**  – individuals with a disease who incorrectly receive a negative screening result

**False positive** – individuals without a disease who incorrectly receive a positive screening result

**Incidence** – the number of instances of disease during a given period in a specified population

**Prevalence** – the number of instances of disease in the population at a single point in time

**Sensitivity** – the proportion of individuals with the disease who will correctly receive a positive screen result, that is, the ability of the test to correctly identify individuals with the disease

**Specificity** – the proportion of individuals without the disease who will correctly receive a negative screen result, that is, the ability of the test to correctly exclude individuals without the disease

**True negative** – individuals without a disease who correctly receive a negative screening result

**True positive** – individuals with a disease who correctly receive a positive screening result

Key terms relating to modelling studies are defined below:

**Base case** – the results generated by a model using the default/preferred settings and inputs

**Bias** – a systematic error in one or more aspects of a study, potentially impacting the observed results and corresponding conclusion of the study; the risk of bias can be appraised using appropriate quality assessment tools

**Decision trees** – a type of model that details a pathway of a disease patient as a set of potential outcomes based on an initial decision (e.g. on a specific intervention), with different probabilities assigned to each of the outcomes

**Deterministic sensitivity analysis** – a type of sensitivity analysis based on varying input parameters one by one in order to determine to what extent the change in individual inputs has an impact on the output values

**Probabilistic sensitivity analysis** – a type of sensitivity analysis based on varying all input parameters at once for a pre-specified number of iterative simulations in order to determine the spread of possible model outputs and evaluate the level of confidence in the model outcome

**Scenarios**  – alternative situations that a model can generate results for (e.g. based on different inputs or assumptions), as opposed to the default base case settings of the model

**Sensitivity analyses** – a group of methods which try and test how reliable the results of a model are; this is done by making small changes to model inputs and measuring the effect on the key outputs (also see deterministic and probabilistic sensitivity analysis)
