# Supplementary material for: The impact of ultrasound-based antenatal screening strategies to detect vasa praevia in the United Kingdom: An exploratory study using decision analytic modelling methods
Source: PLoS One. 2022 Dec 20;17(12):e0279229. doi: 10.1371/journal.pone.0279229 (PMC9767376; doi:10.1371/journal.pone.0279229)
Supplement: Attachment — Submitted filename: Vasa-Praevia(5).pdf [file pone.0279229.s009.pdf]

The Royal Australian and New Zealand College of Obstetricians and Gynaecologists
Excellence in Women's Health

# Vasa praevia

This statement has been developed and reviewed by the Women's Health Committee and approved by the RANZCOG Board and Council.

A list of Women's Health Committee Members can be found in Appendix A.

Disclosure statements have been received from all members of this committee.

Disclaimer This information is intended to provide general advice to practitioners. This information should not be relied on as a substitute for proper assessment with respect to the particular circumstances of each case and the needs of any patient. This document reflects emerging clinical and scientific advances as of the date issued and is subject to change. The document has been prepared having regard to general circumstances.

First endorsed by RANZCOG: July 2012
Current: November 2019
Review due: November 2022

Objectives: To provide advice on the screening for, diagnosis and management of vasa praevia.

Target audience: Health professionals providing maternity care.

Values: The evidence was reviewed by the Women's Health Committee (RANZCOG), and applied to local factors relating to Australia and New Zealand.

Validation: This statement was compared with RCOG and SOGC guidance on this topic.

Background: This statement was first developed by Women's Health Committee in July 2012 and reviewed in November 2019.

Funding: This statement was developed by RANZCOG and there are no relevant financial disclosures.

# Table of contents


1. Plain language summary ........ 3
2. Summary of recommendations ........ 3
3. Introduction ........ 4
4. Discussion and recommendations ........ 4
   4.1 Perinatal mortality and morbidity ........ 4
   4.2 Prenatal diagnosis of vasa praevia ........ 4
   4.3 Screening for vasa praevia ........ 5
      4.3.1 Universal screening ........ 5
      4.3.1.1 Transvaginal imaging ........ 5
      4.3.1.2 Transabdominal imaging ........ 5
      4.3.2 Risk factors ........ 6
      4.3.3 Targeted screening ........ 6
   4.4 Management following antenatal diagnosis ........ 7
   4.5 Emergency management ........ 7
5. Conclusion ........ 8
6. References ........ 8
7. Links to other College statements ........ 9
8. Patient information ........ 9

Appendix A Women's Health Committee Membership ........ 10

Appendix B Overview of the development and review process for this statement ........ 10

Appendix C Full Disclaimer ........ 12

# 1. Plain language summary

Vasa praevia is a rare but potentially serious condition in which blood vessels carrying blood between the placenta and the baby cross over the cervix. These vessels may bleed if the woman goes into labour, if the waters break, or if the cervix opens. Ultrasound examination of women who have risk factors is recommended to determine if vasa praevia is present. If it is found, then special care must be taken in managing the pregnancy and planning the birth.

# 2. Summary of recommendations

| Recommendation 1 | Grade |
|---|---|
| Transvaginal ultrasound using colour and pulse-wave Doppler to evaluate the internal os and lower uterine segment is the most accurate means to diagnose vasa praevia. | Evidence-based recommendation<br>D |
| **Recommendation 2** | **Grade** |
| Vasa praevia should be diagnosed when a fetal vessel, or vessels,are seen either traversing the region of the internal os, or located within 2cm from the internal os. | Good Practice Point |
| **Recommendation 3** | **Grade** |
| Routine screening for vasa praevia of singleton pregnancies with transvaginal ultrasound is not recommended. [4,5] | Consensus-based recommendation |
| **Recommendation 4** | **Grade** |
| Where possible, documentation of placental cord insertion at the routine mid-trimester scan using transabdominal ultrasound and colour Doppler is recommended. | Consensus-based recommendation |
| **Recommendation 5** | **Grade** |
| The presence of a velamentous cord insertion, succenturiate lobe, placenta praevia, IVF pregnancy or other risk factors associated with vasa praevia at the mid-trimester scan should prompt further evaluation by appropriately trained personnel that may include a transvaginal scan. | Good Practice Point |
| **Recommendation 6** | **Grade** |
| Consider admitting women with prenatally diagnosed vasa praevia to a hospital from around 30-32 weeks gestation until delivery and administration of corticosteroids for lung maturity. This will depend on the presence of associated risk factors and proximity to centres with appropriate neonatal facilities. | Good Practice Point |
| **Recommendation 7** | **Grade** |
| Consider delivery between 34-36 weeks gestation. [5] | D |
| **Recommendation 8** | **Grade** |
| Vasa praevia should be suspected in pregnancies with fresh vaginal bleeding (+/- membrane rupture) and acute fetal compromise with heart rate abnormalities such as progressive tachycardia, prolonged bradycardia or sinusoidal pattern. Don't delay delivery to confirm diagnosis.[5] | Good Practice Point |
| **Recommendation 9** | **Grade** |
| In the presence of bleeding from suspected vasa praevia, delivery by urgent Caesarean section is appropriate with paediatric support for neonatal resuscitation including possible immediate transfusion with O Rh negative | Good Practice Point |

| | |
|---|---|
| blood. Those responsible for care of the neonate should be advised of the suspected fetal blood loss prior to caesarean section. | |

## 3. Introduction

Vasa praevia occurs when exposed fetal vessels within the amniotic membranes cover or are in close proximity to the internal cervical os. There are two types: Type 1 vasa praevia occurs with velamentous insertion of the umbilical cord into the placenta and Type II vasa praevia occurs with a velamentous fetal vessel connecting the placenta to a succenturiate placental lobe.[1] Fetal death from rapid exsanguination may occur if the exposed fetal vessels rupture at the time of spontaneous or artificial membrane rupture.

The Australian Maternity Outcomes Surveillance System (AMOSS) has conducted the first national population based study to describe the incidence, management and outcomes of vasa praevia in Australia.[6] When it is published, this study will be used to further inform guidelines regarding the optimal management of pregnancies complicated by vasa praevia. The aim of management is to minimise emergency presentations with antepartum haemorrhage of fetal origin and so reduce perinatal mortality and morbidity from vasa praevia.

Although uncommon the reported incidence of vasa praevia is 1 in 2500[2], (1 in 5000 AMOSS Study). Current evidence indicates vastly improved outcomes associated with antenatal diagnosis.[3] However, achieving prenatal diagnosis is not clear cut as there are pitfalls with the ultrasound diagnosis of vasa praevia and the role of ultrasound screening in pregnancy lacks a robust evidence base. When vasa praevia is diagnosed antenatally, management guidelines based on the best available evidence recommend strategies to improve outcomes.[4,5]

## 4. Discussion and recommendations

### 4.1 Perinatal mortality and morbidity

Diagnosing vasa praevia prenatally is associated with significantly improved perinatal survival. One multicentre retrospective cohort study of 155 cases of vasa praevia reported the overall perinatal mortality as 36%. Compared with a 97% (59/61) infant survival in the cases diagnosed prenatally, the survival in the cases not diagnosed prenatally was 44% (41/94).On logistic regression analysis, the variable "prenatal diagnosis" was a significant predictor of survival (OR 102.9; 95%CI 16.2 to 638.3; $p<0.001$).[3] Corresponding neonatal transfusion rates between prenatally diagnosed and undiagnosed cases were 3.4% and 58.5%, respectively ($p<0.001$).[3] In another 19 cases in a single centre retrospective cohort study, there were no cases (0/10) of cord blood pH <7 in vasa praevia cases diagnosed prenatally, but 33.3% (3/9) with cord blood pH <7 when not diagnosed prenatally.[7]

A more recent prospective, population-based cohort study using the Australian Maternity Outcomes Surveillance System (AMOSS) found there were no perinatal deaths in the 58 cases diagnosed prenatally out of the 63 cases confirmed with vasa praevia at birth.[2]

### 4.2 Prenatal diagnosis of vasa praevia

Vasa praevia was first diagnosed antenatally by real-time ultrasound in 1987[8] and colour Doppler in 1990.[9] Abdominal wall scarring, obesity, or an empty maternal bladder may compromise transabdominal assessment of the lower segment.[10,11] A recent systematic review of the accuracy of ultrasound in diagnosing vasa praevia, concluded that ultrasound diagnosis is highest when performed transvaginally in combination with colour Doppler. Overall in the eight included studies, the antenatal detection rates varied from 53 to 100%. However, analysis of the only two prospective studies included, showed that all cases of vasa

praevia were detected (sensitivity 100%) with a specificity of 99-99.8% when transvaginal colour Doppler was performed.[12]

It must be recognised that not all cases of vasa praevia can be diagnosed antenatally.[11] Transvaginally the direction of the fetal vessels may inhibit diagnosis. False positives are not uncommon and can arise from motion artefacts, umbilical cord (funic) presentation or a marginal placental sinus.[10,11,13] Prenatal diagnosis appears most effective during mid-pregnancy (18-24 weeks of gestation) but needs to be confirmed at 30 to 32 weeks of gestation.[12]

Using a transvaginal approach to image the internal cervical os and lower uterine segment, the diagnostic criteria for VP includes:

- Visualising aberrant linear or tubular echolucent structures with 2D imaging[10]
- Demonstrating blood flow in these structures using colour or power Doppler[13]
- Demonstrating umbilical arterial/venous Doppler waveforms using pulse wave Doppler[4]
- Aberrant vessels located over or within 2cm of the internal os attached to the inner perimeter of the fetal membranes[14]

| Recommendation 1 | Grade |
|---|---|
| Transvaginal ultrasound using colour and pulse-wave Doppler to evaluate the internal os and lower uterine segment is the most accurate means to diagnose vasa praevia. | Evidence-based recommendation<br><br>D |
| **Recommendation 2** | **Grade** |
| Vasa praevia should be diagnosed when a fetal vessel, or vessels,are seen either traversing the region of the internal os, or located within 2cm from the internal os. | Good Practice Point |

## 4.3 Screening for vasa praevia

There is no consensus from the international obstetric community about screening for prenatal detection of vasa praevia. Only the Society of Obstetricians and Gynaecologists of Canada (SOGC) and Royal College of Obstetricians and Gynaecologists (RCOG) have published practice guidelines on the diagnosis and management of vasa praevia and they differ in their recommendations for screening.[4,5] Because there are no large prospective trials, screening strategies rely on data combined from many small case series. Different strategies have been considered and require informed debate.

### *4.3.1 Universal screening*

#### *4.3.1.1 Transvaginal imaging*

Vasa praevia is uncommon and there is no robust evidence that universal screening of the general population using routine transvaginal imaging would be accurate, practical or improve perinatal outcomes.[11] In addition, a cost-utility analysis performed in Canada in 2010 concluded that routine transvaginal screening for vasa praevia in singleton pregnancies at the mid trimester obstetric scan is not cost-effective.[15] Concordantly, universal screening by routine transvaginal ultrasound is not recommended by either the SOGC or the RCOG.[4,5]

#### *4.3.1.2 Transabdominal imaging*

Approximately half of all cases of vasa praevia occur in the setting of a velamentous cord insertion. Therefore, routine screening of all pregnancies by transabdominal ultrasound to ascertain either a velamentous insertion of the umbilical cord into the placenta or the presence of a multi-lobed placenta has been proposed. Velamentous placental cord insertion occurs in approximately 1% of singleton pregnancies.[16] Detection would enable evaluation of the lower segment for the presence

of a vasa praevia that occurs in only 2% of velamentous placental cord insertions[17] (see Targeted screening).

The feasibility of assessing the placental cord insertion has been studied in both the first and second trimesters with no advantage being found in first trimester assessment. Prospective studies at the midtrimester anomaly scan have demonstrated that placental cord insertions are quickly identifiable using colour Doppler without further training in ≥ 99% pregnancies.[16,18] The accuracy of routine midtrimester screening to detect velamentous cord insertions have been reported in a prospective study of 3446 cases with a sensitivity of 62.5%, positive predictive value of 100% and negative predictive value of 99.5%.[19]

### *4.3.2 Risk factors*

In addition to its association with velamentous cord insertion, vasa praevia is also associated with bi-lobed and succenturiate placental abnormalities, placenta praevia, a history of a low-lying placenta in the second trimester, IVF and multiple pregnancy.[2] A retrospective analysis of these risk factors in 12063 deliveries from a single centre that routinely screens for vasa praevia, determined which of these variables were independent risk factors for vasa praevia. Second trimester placenta praevia, with an odds ratio of 22.86 (95%CI 5.57-93.78) and bi-lobed or succenturiate placentas, with an odds ratio of 22.11 (95%CI 1.92-253.84) are the most significant risk factors.[20] IVF is a significant risk factor with an odds ratio of 7.75 (95%CI 1.99-30.10) and increases the incidence of vasa praevia to as high as 1 in 200.[10,21] Multiple pregnancy was not an independent risk factor.[20]

### *4.3.3 Targeted screening*

Targeted ultrasound screening for vasa praevia is advocated by many authors. [3,13,20,22] A comprehensive literature review identified 28 cases of prenatally diagnosed vasa praevia in 17 publications over 16 years and has advocated screening ultrasound and suggested the following screening algorithm.[13] All pregnancies are screened abdominally at the midtrimester ultrasound for the location of the lower margin of the placenta and the location of the placental cord insertion. If these are greater than 2cm from the internal os, no further assessment is required. If there is a low lying, succenturiate or bi-lobed placenta, a velamentous cord insertion or an IVF or multiple pregnancy, then an abdominal scan of the cervix is performed with colour Doppler. If there are any suspicious findings or poor visualisation, then a transvaginal scan is performed to optimise the diagnosis. Concordant with this algorithm, the 2010 cost-utility analysis was also in favour of targeted screening. This study concludes that the use of transabdominal colour Doppler at all singleton mid trimester ultrasound scans with targeted transvaginal scans in the presence of velamentous cord insertions, placental abnormalities or IVF pregnancies is cost effective. Screening all twin pregnancies with transvaginal ultrasound was found to be cost-effective as the QALY-gained was not different between twins resulting from IVF and all twins.[15] SOGC recommends targeted transvaginal screening in the presence of risk factors[3]. The RCOG does not recommend any screening for vasa praevia but acknowledges that some centres, based on their case mix, screening those with risk factors may be justifiable.[5]

| Recommendation 3 | Grade |
|---|---|
| Routine screening for vasa praevia_of singleton pregnancies with transvaginal ultrasound is not recommended. [4,5] | Consensus-based recommendation |
| **Recommendation 4** | **Grade** |
| Where possible, documentation of placental cord insertion at the routine mid-trimester scan using transabdominal ultrasound and colour Doppler is recommended. | Consensus-based recommendation |
| **Recommendation 5** | **Grade** |
| The presence of a velamentous cord insertion, succenturiate lobe, placenta praevia, IVF pregnancy or other risk factors associated with vasa praevia at the mid-trimester scan should prompt further evaluation by appropriately trained personnel that may include a transvaginal scan. | Good Practice Point |

### 4.4 Management following antenatal diagnosis

There are no clinical trials to inform the optimal management in cases of confirmed vasa praevia and because the severity of the outcome, are not ethically justifiable.[11] Consequently, the best management strategies are based on retrospective case series and consensus views. For confirmed cases of vasa praevia with no bleeding, both SOGC and RCOG clinical guidelines suggest the following management[4,5]

- Admission to hospital from 30 weeks gestation until the time of delivery to expedite urgent emergency delivery in the event of membrane rupture, vaginal bleeding or preterm labour;
- Administration of corticosteroids for fetal lung maturation in anticipation of preterm delivery;
- Admission and delivery in a hospital with paediatric expertise and appropriate level of neonatal care;
- Delivery by elective caesarean section prior to the onset of labour.

Other options include:

- Outpatient management of select asymptomatic singleton cases with a long,closed cervix on serial transvaginal ultrasound scans and a negative fetal fibronectin[3,22] This option is supported by a retrospective cohort study published in 2013 which reported a 4% risk of preterm emergency delivery in singleton pregnancies diagnosed prenatally.[23]
- The optimal gestation at which to admit patients with a prenatal diagnosis of vasa praevia may depend on the circumstances of the patient and availability of the appropriate inpatient facilities.
- Transvaginal ultrasound with colour Doppler to map fetal vessels preoperatively to avoid iatrogenic laceration and intraoperative fetal haemorrhage.

The optimal gestation at which to deliver is balanced by the risks of perinatal mortality and morbidity from ruptured vasa praevia and the risks associated with iatrogenic prematurity. The largest published series of prenatally diagnosed cases reported a mean gestational age at delivery of 34.9 +/- 2.5 weeks but 27.9% were emergency caesarean sections for bleeding, labour or ruptured membranes.[3] This study demonstrated on logistic regression analysis, the variable "gestational age at delivery" was the other significant predictor of survival besides "prenatal diagnosis" (OR 0.77; 95%CI 0.64 to 0.93; $p<0.01$).[3] The mean gestation at delivery of undiagnosed cases, and that associated with a 56% perinatal mortality, was 38.2 +/-2.1 weeks. These authors claim that delivery at later gestational ages may negate the benefit of prenatal diagnosis.[3]

To further inform practise, a decision analysis published with 11 strategies for timing delivery weekly from 32 to 39 weeks gestation factored in lifetime risks of death, respiratory distress syndrome, cerebral palsy and neurodevelopmental disability. This indicated that following prenatal diagnosis of vasa praevia, the preferred timing for elective caesarean section is 34 or 35 weeks gestation under most but not all circumstances. Under no clinical circumstances was there a benefit to be gained by expectant management beyond 37 weeks gestation.[24]

| Recommendation 6 | Grade |
|---|---|
| Consider admitting women with prenatally diagnosed vasa praevia to a hospital with appropriate neonatal facilities from around 30 weeks gestation until delivery and administration of corticosteroids for lung maturity. | Good Practice Point |
| **Recommendation 7** | **Grade** |
| Consider delivery between 34-36 weeks gestation. [5] | D |

### 4.5 Emergency management

While antenatal diagnosis optimises outcome among women with known vasa praevia, undiagnosed cases will still occur. Vasa praevia should be suspected in pregnancies with fresh vaginal bleeding (+/- membrane rupture) and acute fetal compromise with heart rate abnormalities such as progressive tachycardia, prolonged bradycardia, sinusoidal pattern or fetal death.[3,10,11] While bedside tests are available to establish if vaginal bleeding is of fetal origin, accessing these tests is usually too slow of be of any clinical use.[2] Because of the small fetal blood volume, the loss of relatively small amounts of blood may lead to fetal shock.[2] The prognosis associated with bleeding from a fetal vessel is poor. Urgent emergency Caesarean section to effect rapid delivery and paediatric support for neonatal resuscitation including immediate transfusion with O Rh negative blood may be lifesaving.[3] The rate of transfusion in surviving neonates of vasa praevia not diagnosed antenatally is reported as 58.5.%.[3] Neonatal paediatricians need to be advised

of the suspected diagnosis of bleeding vasa praevia before Caesarean section so that they can make the required preparations to resuscitate an infant whose circulating blood volume is depleted.

| Recommendation 8 | Grade |
|---|---|
| Vasa praevia should be suspected in pregnancies with fresh vaginal bleeding (+/- membrane rupture) and acute fetal compromise with heart rate abnormalities such as progressive tachycardia, prolonged bradycardia or sinusoidal pattern. Don't delay delivery to confirm diagnosis.[5] | Consensus-based recommendation |
| **Recommendation 9** | **Grade** |
| In the presence of bleeding from suspected vasa praevia, delivery by urgent Caesarean section is appropriate with paediatric support for neonatal resuscitation including possible immediate transfusion with O Rh negative blood. Those responsible for care of the neonate should be advised of the suspected fetal blood loss prior to caesarean section. | Good Practice Point |

## 5. Conclusion

Vasa praevia is an uncommon but potentially life-threatening condition for the fetus/neonate. Perinatal outcomes improve significantly when antenatal diagnosis enables planned management that includes elective Caesarean section by 35 weeks gestation before the onset of labour. Universal screening to locate the placental cord insertion at the routine mid trimester scan enables further assessment of a velamentous cord insertion by appropriately trained personnel using colour Doppler and transvaginal imaging. In the event of an emergency Caesarean section for a suspected vasa praevia, paediatricians should be informed and prepared for immediate neonatal transfusion with O Rh negative blood in the event of haemorrhagic shock.

Placental histopathology is also recommended in cases of stillbirth and neonatal death.[5]

## 6. References


1. Catanzarite V, Maida C, Thomas W, Mendoza A, Stanco L, Piacquadio KM. Prenatal sonographic diagnosis of vasa previa: ultrasound findings and obstetric outcome in ten cases. Ultrasound Obstet Gynecol 2001; 18(2): 109-115.
2. Oyelese KO, Turner M, Lees C, Campbell S. Vasa previa: an avoidable tragedy. Obstet Gynecol Surv 1999; 54: 138-145.
3. Oyelese Y, Catanzarite V, Prefumo F, Lashley S, Schachter M, Tovbin Y, Goldstein V, Smulian JC. Vasa previa: the impact of prenatal diagnosis on outcomes. Obstet Gynecol 2004; 103 (5 Pt 1): 937-42.
4. Gagnon R. Guidelines for the management of vasa previa. J Obstst Gynecol Can 2017 31: 748-753
5. Jauniaux, ERM, Alfirevic, Z, Bhide, AG, Burton, GJ, Collins, SL, Silver, R on behalf of the Royal College of Obstetricians and Gynaecologists. Vasa praevia: diagnosis and management. Green-top Guideline No. 27b. BJOG 2018
6. Vasa previa AMOSS available at: http://www.amoss.com.au/content/vasa-praevia
7. Smorgick N, Tovbin Y, Ushakov F, Vaknin Z, Barzilay B, Herman A, Maymon R. Is neonatal risk from vasa previa preventable? The 20-year experience from a single medical center. J Clin Ultrasound 2010; 38:118-122.
8. Gianopoulos J, Carver T, Tomich PG, Karlman R, Gadwood K. Diagnosis of vasa previa with ultrasonography. Obstet Gynecol 1987; 69: 488-491
9. Nelson LH, Malone PJ, King m. Diagnosis of vasa previa with transvaginal and colour Doppler

ultrasound. Obstet Gynecol. 1990; 76506.
10. Lee W, Lee VL, Kirk JS, Sloan CT, Smith RS, Comstock CH. Vasa previa: prenatal diagnosis, natural evolution, and clinical outcome. Obstet Gynecol 2000; 95: 572-6
11. Nishtar A, Wood PL. Is it time to actively look for vas previa? J Obstet Gynecol 2012: 32:413-18
12. Ruiter L, Kok N, Limpens J, Derks JB, De Graaf IM, Mol BJW, Pajkrt E. Systematic review of accuracy of ultrasound in the diagnosis of vasa previa. Ultrasound Obstet Gynecol 2015; 45: 516-522
13. Derbala Y, Grochai F, Jeanty P. Vasa previa. J Perinat Med 2007; 1 2-13.
14. Rebarber A, Dolin C, Fox NS, Klauser CK, Saltzman DH, Roman AS. Natural history of vasa previa across gestation using a screening tool. J Ultrasound Med 2014; 33: 141-147.
15. Cipriano LE, Barth WH Jr, Zaric GS. The cost-effectiveness of targeted or universal screening for vasa praevia at 18-20 weeks of gestation in Ontario. BJOG 2010; 117 (9): 1108-18. Epub 2010 Jun 18.
16. Sepulveda W, Rojas I, Robert JA, Schnapp C, Alcalde JL. Prenatal detection of velementous insertion of the umbilical cord : a prospective color Doppler ultrasound study. Ultrasound Obstet Gynecol 2003; 21: 564-569.
17. Quek SP, Tan KL. Vasa previa. Aust J Obstet Gynecol 1972; 12: 206-9.
18. Nomiyama M, Toyota Y, Kawano H. Antenatal diagnosis of velementous cord insertion and vasa previa with color Doppler imaging. Ultrasound Obstet Gynecol 1998; 12: 426-429
19. Hasegawa J, Matsuoka R, Itchizuka K, Sekizawa A, Farina A, Okai t. Velamentous cord insertion into the lower third of the uterus is associated with intrapartum fetal heart rate abnormalities. Ultrasound Obstet Gynecol 2006; 27: 425-429.
20. Baulies S, Maiz N, Muñoz A, Torrents M, Echevarría M, Serra B. Prenatal ultrasound diagnosis of vasa praevia and analysis of risk factors. Prenat Diagn 2007; 27 (7): 595-9.
21. Schachter M, Tovbin Y, Arieli S, Friedler S, Ron-El R, Sherman D. In vitro fertilization is a risk factor for vasa previa. Fertil Steril 2002; 78; 642-3.
22. Donnolly N, Halliday LE, Oyelese Y. Vasa Previa: a descriptive review of existing literature and the evolving role of ultrasound in prenatal screening. Aust J Ultrasound Med 2013; 16: 71-6.
23. Bronsteen R, Whitten A, Balasubramanian M et al. Vasa Previa. Obstet Gynecol 2013; 122:352-7.
24. Robinson BK & Grobman WA. Effectiveness of timing strategies for delivery of individuals with vasa previa. Obstet Gynecol 2011; 117: 542-9.
25. Sullivan EA, Javid N, Duncombe G, Li Z, Cincotta R, et al. Vasa previa Diagnosis, Clinical Practice, and Outcomes in Australia. Obstet Gynecol 2017; 130:591-8. (AMOSS STUDY)

## 7. Links to other College statements

Measurement of Cervical Length in Pregnancy (C-Obs 27)
https://ranzcog.edu.au/RANZCOG_SITE/media/RANZCOG-MEDIA/Women%27s%20Health/Statement%20and%20guidelines/Clinical-Obstetrics/Measurement-of-cervical-length-for-prediction-of-preterm-birth(C-Obs-27)-Review-July-2017.pdf?ext=.pdf

Evidence-based Medicine, Obstetrics and Gynaecology (C-Gen 15)
https://www.ranzcog.edu.au/RANZCOG_SITE/media/RANZCOG-MEDIA/Women%27s%20Health/Statement%20and%20guidelines/Clinical%20-%20General/Evidence-based-medicine,-Obstetrics-and-Gynaecology-(C-Gen-15)-Review-March-2016.pdf?ext=.pdf

## 8. Patient information

A range of RANZCOG Patient Information Pamphlets can be ordered via:

https://www.ranzcog.edu.au/Womens-Health/Patient-Information-Guides/Patient-Information-Pamphlets

# Appendices

## Appendix A Women's Health Committee Membership

| Name | Position on Committee |
|---|---|
| Professor Yee Leung | Chair and Board Member |
| Dr Gillian Gibson | Deputy Chair, Gynaecology |
| Dr Scott White | Deputy Chair, Obstetrics and Subspecialties Representative |
| Associate Professor Ian Pettigrew | Member and EAC Representative |
| Dr Kristy Milward | Member and Councillor |
| Dr Will Milford | Member and Councillor |
| Dr Frank O'Keeffe | Member and Councillor |
| Professor Steve Robson | |
| Professor Sue Walker | Member |
| Dr Roy Watson | Member and Councillor |
| Dr Susan Fleming | Member and Councillor |
| Dr Sue Belgrave | Member and Councillor |
| Dr Marilyn Clarke | ATSI Representative |
| Associate Professor Kirsten Black | Member |
| Dr Thangeswaran Rudra | Member |
| Dr Nisha Khot | Member and SIMG Representative |
| Dr Judith Gardiner | Diplomate Representative |
| Dr Angela Brown | Midwifery Representative, Australia |
| Ms Adrienne Priday | Midwifery Representative, New Zealand |
| Ms Ann Jorgensen | Community Representative |
| Dr Rebecca Mackenzie-Proctor | Trainee Representative |
| Dr Leigh Duncan | Maori Representative |
| Prof Caroline De Costa | Co-opted member (ANZJOG member) |
| Dr Christine Sammartino | Observer |

## Appendix B Overview of the development and review process for this statement

i. *Steps in developing and updating this statement*

This statement was originally developed in July 2012 and was most recently reviewed in November 2019. The Women's Health Committee carried out the following steps in reviewing this statement:

- Declarations of interest were sought from all members prior to reviewing this statement.
- Structured clinical questions were developed and agreed upon.
- An updated literature search to answer the clinical questions was undertaken.
- At the November 2019 face-to-face committee meeting, the existing consensus-based recommendations were reviewed and updated (where appropriate) based on the available body of evidence and clinical expertise. Recommendations were graded as set out below in Appendix B part iii)

ii. *Declaration of interest process and management*

Declaring interests is essential in order to prevent any potential conflict between the private interests of members, and their duties as part of the Women's Health Committee.

A declaration of interest form specific to guidelines and statements was developed by RANZCOG and approved by the RANZCOG Board in September 2012. The Women's Health Committee members were required to declare their relevant interests in writing on this form prior to participating in the review of this statement.

Members were required to update their information as soon as they become aware of any changes to their interests and there was also a standing agenda item at each meeting where declarations of interest were called for and recorded as part of the meeting minutes.

There were no significant real or perceived conflicts of interest that required management during the process of updating this statement.

*iii. Grading of recommendations*

Each recommendation in this College statement is given an overall grade as per the table below, based on the National Health and Medical Research Council (NHMRC) Levels of Evidence and Grades of Recommendations for Developers of Guidelines. Where no robust evidence was available but there was sufficient consensus within the Women's Health Committee, consensus-based recommendations were developed or existing ones updated and are identifiable as such. Consensus-based recommendations were agreed to by the entire committee. Good Practice Notes are highlighted throughout and provide practical guidance to facilitate implementation. These were also developed through consensus of the entire committee.

| Recommendation category | | Description |
|---|---|---|
| Evidence-based | A | Body of evidence can be trusted to guide practice |
| | B | Body of evidence can be trusted to guide practice in most situations |
| | C | Body of evidence provides some support for recommendation(s) but care should be taken in its application |
| | D | The body of evidence is weak and the recommendation must be applied with caution |
| Consensus-based | | Recommendation based on clinical opinion and expertise as insufficient evidence available |
| Good Practice Note | | Practical advice and information based on clinical opinion and expertise |

## Appendix C Full Disclaimer

This information is intended to provide general advice to practitioners, and should not be relied on as a substitute for proper assessment with respect to the particular circumstances of each case and the needs of any patient.

This information has been prepared having regard to general circumstances. It is the responsibility of each practitioner to have regard to the particular circumstances of each case. Clinical management should be responsive to the needs of the individual patient and the particular circumstances of each case.

This information has been prepared having regard to the information available at the time of its preparation, and each practitioner should have regard to relevant information, research or material which may have been published or become available subsequently.

Whilst the College endeavours to ensure that information is accurate and current at the time of preparation, it takes no responsibility for matters arising from changed circumstances or information or material that may have become subsequently available.
