# Supplementary material for: The impact of ultrasound-based antenatal screening strategies to detect vasa praevia in the United Kingdom: An exploratory study using decision analytic modelling methods
Source: PLoS One. 2022 Dec 20;17(12):e0279229. doi: 10.1371/journal.pone.0279229 (PMC9767376; doi:10.1371/journal.pone.0279229)
Supplement: Attachment — Submitted filename: Response to Reviewers.docx [file pone.0279229.s008.docx]

**Reviewer 1**

***Question/Comment:*** *The authors carried out an exploratory study using decision analytic modelling methods examining various targeted screening methods for vasa previa in the United Kingdom. They evaluate screening for vasa previa in a hypothetical UK population in patients with low-lying placentas, velamentous cord insertions, and pregnancies resulting from IVF, and also with no screening. They find that examining VCI based screening and low-lying placentas resulted in the highest detection rates and lowest rates of perinatal death.*

*The manuscript is well-written and the methodology is sound. The conclusions are supported by the methods. However, any model is only as good as the assumptions put into it..*

**Response:** We thank the reviewer for this comment and the positive assessment of the presented manuscript. We agree with the raised point that the reliability of a model is largely dependent on the reliability of the underlying assumptions. One of the reasons we undertook this study was to draw attention to the need for research in this important area, and we hope that the inclusion of a full set of sensitivity and relevant scenario analyses as part of the presented results will also go towards accounting for the reliance on assumptions and limited available data.

Already with regards to characterisation of the different modelled strategies (which are also discussed in more detail further below), the reviewer might note that one of the strategies (velamentous cord insertion (VCI)) has been designed to represent an universal, or population, screening strategy and another (IVF-based) strategy has been described as a representation of risk assessment in routine clinical care.

***Question/Comment:*** *Perhaps a flaw of this study, and of several others is the question: “What constitutes “screening” for vasa previa?” I suggest that the authors define clearly what is meant by “screening” for vasa previa. A flaw of this study and of the UK screening evaluations at present is that it makes the assumption that screening involves transvaginal ultrasound with Doppler. This is more diagnosis, and the authors continue perpetuating the myth that vasa previa cannot be routinely screened for (which could be done via routinely identifying placental cord insertion and a Doppler sweep of the lower uterine segment.*

*How do THESE authors define “screening” for vasa previa? Perhaps the only thing I find in their manuscript is “recall at 32 weeks to perform further TAS to confirm the presence of VP (representing a population screening strategy based on a risk factor not currently reported in UK practice)” (Line 118).*

**Response:** Many thanks for this comment. We have tried to address the request to state an overall definition of screening by adding this to the relevant paragraph in the Introduction. We have also included further argumentation (both within the Introduction as well as the description of the different strategies within the Methods section) to highlighted the reasoning behind drawing a distinction between the VCI strategy, as an example of universal/population screening, and the LLP strategy as an example of targeted screening (please note that we have also included an additional reference for this term/concept in the form of a recently published review by Bobrowska et al. 2022).

Interestingly, available guidance by the Royal Australian and New Zealand College of Obstetricians and Gynaecologists (RANZCOG) also refers to a universal screening strategy based on the detection of VCI (followed by confirmation including TVS); we take this as some confirmation of our use of the term.

We acknowledge that, while concepts and categories aim to assist orientation to problems, they are always also open to debate. We hope that the amendments we have provided will help the reviewer, as well as the eventual reader, to reflect on the conceptual differences between the strategies.

Regarding the point about transvaginal sonography (TVS), we would like to emphasise that we agree with the reviewer that TVS is a diagnostic procedure in this context and that abdominal ultrasound would constitute the screening procedure. However, it is important to consider the throughput from screening to diagnostic services and one of the aims of the paper is to also explore this aspect of the different strategies described in the model. We would also like to refer again to the available RANZCOG guidance as another example, outside of the UK context, that considers a confirmatory step (including TVS) as part of their recommended universal screening strategy, providing additional support for the inclusion of this step as part of our modelled strategy (which was initially informed and validated through consultation with a selection of relevant UK experts).

***Question/Comment:*** *The authors also do not examine the role of universal screening as part of their study. Wouldn’t it be worth seeing how universal screening performed in this context? Perhaps tied into this is the question above: “What constitutes screening for vasa previa?*

**Response:** Many thanks for this comment. We hope that we have addressed this concern as part of the above response and the corresponding amendments to the manuscript.

In keeping with the RANZCOG guideline, we consider the VCI-based strategy to be a suitable universal, or population, screening strategy, whilst also recognising that this may be open to debate.

***Question/Comment:*** *The authors state in line 122 “there is no independently significant association between multiple pregnancies and VP incidence.” Is this actually true?*

**Response:** Many thanks for this query. The statement in question is informed by a thorough literature review commissioned by the UK NSC, which is further corroborated by the findings of a widely cited systematic literature review/meta-analysis performed by Ruiter et al. 2016 (now also added as supporting reference for this sentence in the manuscript).

However, we acknowledge that both publications further state that these findings appear to be in contradiction to previous studies (e.g. Ruiter et al. mention that “most authors of case reports report multiple pregnancies as a risk indicator for VP; however, our data fail to support this finding”). As such, we have included some additional wording to the relevant sentence, to specify that the focus on singleton pregnancies is a simplifying assumption based on findings indicating no significant association between multiple pregnancies and VP incidence.

***Question/Comment:*** *The UK NSC estimate of the incidence of VP of 0.03% of pregnancies is likely an underestimation. The authors have addressed this. However, the UKOSS may have its own inherent flaws.*

**Response:** Many thanks for this observation. We fully agree with the stated issue of obtaining robust relevant incidence estimates for this rare condition to use as input in the model. However, we hope that this is sufficiently accounted for by the consideration of relevant scenario analyses, as well as the already included discussion of the general parameter uncertainty (pointing out the prevalence of VP in particular) and the UKOSS study specifically (in the context of diagnostic criteria and the possible discrepancy between ultrasound-detected and clinically presenting VP).

***Question/Comment:*** *An important finding is that clearly using IVF alone as the screening for vasa previa will detect few cases, and lead to the highest number of deaths from VP.*

**Response:** Many thanks, we agree with this comment and the assessment of the results for the IVF-based strategy in particular.
