# Supplementary material for: The impact of ultrasound-based antenatal screening strategies to detect vasa praevia in the United Kingdom: An exploratory study using decision analytic modelling methods
Source: PLoS One. 2022 Dec 20;17(12):e0279229. doi: 10.1371/journal.pone.0279229 (PMC9767376; doi:10.1371/journal.pone.0279229)
Supplement: Attachment — Submitted filename: Response to Reviewers.docx [file pone.0279229.s010.docx]

**Editor**

***Question/Comment:*** *You have referred to the Australia and New Zealand guidelines. These guidelines do recommend identification of placental cord insertion where feasible. Kindly address this in your revision.*

**Response:** As suggested, we have now included additional wording in the Introduction to make explicit reference to the RANZCOG guideline, as an example of available guidance in the context of VCI as possible candidate for population screening.

Further to this, we would like to highlight again the already included reference to the RANZCOG guideline as part of the Discussion, when mentioning the existence of guidance that does indeed recommend VCI-based screening.

***Question/Comment:*** *Reference 18 is an oral abstract reference. To my knowledge, this data was never published. Please only include published or accepted manuscripts as references.*

**Response:** We agree with the Editor that the full results of the UKOSS study of vasa praevia do not appear to have been published to date; as such, we would suggest the following amendments to the manuscript:

- Instead of referring to the abstract when discussing the increasing interest in this area as part of the Introduction, we have now included a reference to the UKOSS 2016 annual report which features a section on vasa praevia
- In acknowledgment of the fact that the previously included UKOSS scenario analysis was based on unpublished data obtained through personal communication with the principal investigator (GA), we have now excluded this scenario from the manuscript
- We would suggest to still include the original reference to the abstract when using this to highlight the difference in reported VP mortality rates in the Discussion, as we consider this to be an important point in the discussion of screening and the UKOSS-related statement to be sufficiently supported by the data from the abstract (as published in a supplementary issue of the BJOG; <https://obgyn.onlinelibrary.wiley.com/doi/10.1111/1471-0528.14585>) – however, in order to acknowledge the nature of the reference, we have also included additional wording to highlight that only preliminary results from the UKOSS study are currently available

**Reviewer 1**

***Question/Comment:*** *The authors tackled very important topic of targeted screening and carried out an exploratory study using decision analytic modelling methods examining various targeted screening methods for vasa previa in the United Kingdom. They find that examining VCI based screening and low-lying placentas resulted in the highest detection rates and lowest rates of perinatal death.*

*Although I am not sure regarding the strength of the methodology (although it sounds) this well written manuscript. The conclusions are supported by the methods. However, I agree with other reviewer comment that any model is only as good as the assumptions put into it.. Furthermore, the authors do not examine the role of universal screening and another a flaw of this study is the question: “What constitutes “screening” for vasa previa….*

*Neve the less the authors address the remarks and flaws raised. This is interesting, well written and important study theretofore I recommend accept it.*

**Response:** Thank you very much for your positive feedback and recommendation.

**Reviewer 2**

***Question/Comment:*** *The authors of this UK based study explore targeted screening based on IVF pregnancy, low lying placenta and velamentous cord insertion, compared to no screening. They conclude, using a decision analytical tree method, that screening for VP based on those pregnancies affected by low lying placenta is potentially the most efficient method, avoiding false positive diagnoses and having least impact on the current care pathway.*

*In Lines 93-94 they state that “The overall purpose of this work was to make a practical contribution to the evolving discussion about the antenatal detection of VP in the UK; this was achieved by presenting here an analysis of four possible detection pathways for VP”. This the authors have done using generally reasonable assumptions. However, I am struck that the screening/diagnostic phase of ascertainment of VP in this study is assumed to be by TAS at 32 weeks, when cord insertion and the presence of low lying placenta can be determined with a high degree of certainty at the mid trimester scan (as discussed in reference 7 that the authors cite). Hence what is missing from this analysis is a policy of referral for TAS/TVS at 32 weeks where there is LLP or VCI at the routine mid trimester scan. This would dramatically reduce the number of additional 32 week scans required while very modestly increasing the length of a mid trimester scan and only in those units that don’t already look for cord insertion, as all do already for LLP.*

**Response:** We agree with the Reviewer’s thoughts on the likely merit of including VCI or LLP as part of a possible screening strategy, and we believe this to indeed be covered by the modelled strategies.

Namely, the LLP-based screening strategy involves the routinely performed diagnosis of LLP at the mid-trimester scan as basis for the subsequent referral for TAS/TVS for VP at 32 weeks. Similarly, the VCI-based pathway includes the additional testing for VCI at the mid-trimester scan to indicate the referral for TAS/TVS for VP at 32 weeks in cases where VCI is detected at 18^+0^ to 20^+6^ weeks.

In this context, we would also like to highlight Figure 1 in the submission which provides a flowchart detailing the different steps of the modelled screening pathways, in line with the description above.

***Question/Comment:*** *Lines 99-100: The VP screening model was programmed in Microsoft Excel and used a decision-analytic tree structure to explore the effects of four potential detection pathways in a hypothetical one-year UK pregnancy cohort.*

*-What was the assumed size of this cohort? I cannot see this defined anywhere, except in table 3 862, 785 pregnancies are referred to.*

**Response:** The overall cohort size (i.e. 862,785 pregnancies), which had so far been included in the supplementary Table S1, has now also been added to the list of key inputs in Table 2.

***Question/Comment:*** *Lines 124-126: In the VCI-based pathway, additional testing for VCI and BL/S placenta specifically aimed at establishing the risk of VP would be performed during the 18+0 to 20+6 week scan, with positive detection prompting a recall at 32 weeks to perform further TAS to confirm the presence of VP (representing a population screening strategy based on a risk factor which is sought for the sole purpose of and preventing adverse outcomes from VP, and which is not currently reported in UK practice).*

*-it is not clear what is the screening process: is it a TA scan at 18-20 weeks, followed by a further TAS at 32 weeks, and then a TVS as the diagnostic test? Sorry if I have missed this but if it is submerged in the text somewhere it needs to be clearer, possibly by means of a flow diagram. If the authors are suggesting (as I state above) that LLP and VCI screening can be carried out at 18-20+6 weeks, this would hugely reduce the estimate of additional 32 week TAS scans referred to in Table 3 in the VCI pathway.*

**Response:** We would like to highlight again the flowchart that has been provided as part of Figure 1 in the submission. In line with this, we can confirm that the VCI-based pathway does indeed include proposed testing for VCI (in addition to the routine testing for LLP) as part of the mid-trimester scan at 18^+0^ to 20^+6^ weeks; as described above, detected cases of VCI would then be referred to TAS for VP at 32 weeks (with a final confirmatory TVS for TAS-detected VP at this stage).

We can further confirm that the stated number of referrals to 32-week TAS (for VP) in the VCI-based pathway are modelled as a direct result of the literature-informed incidence of VCI as well as the expected TAS test accuracy for VCI at 18^+0^ to 20^+6^ weeks.

***Question/Comment:*** *Lines 132-133: In all pathways, pregnancies that underwent TAS at 32 weeks were also followed-up by TVS for VP, if VP could not be excluded. Incidental detection of VP across all pregnancies was also accounted for in all four pathways.*

*-TAS cannot exclude VP, TVS can. The normal pathway is TAS for screening and TVS for diagnosis, I am curious to know why the authors suggest that this should be inverted? In other words, once VP is suspected then a TVS should be performed, not another TAS, which would in any event require another TVS.*

**Response:** We agree with the Reviewer regarding the appropriate sequence of screening steps, which we have also further clarified for the modelled strategies in response to the queries above, and apologise for any confusion stemming from the use of “cannot be excluded” for describing the referral to subsequent screening steps.

In response, we have amended the wording where relevant (e.g. as part of the flowchart in Figure 1) to instead say that confirmatory/diagnostic TVS was performed where VP “was suspected” during TAS.

***Question/Comment:*** *Line 149: ….workshops involving six UK clinical experts (GA, BT, NT, AM, EDJ, HG).*

*-How were the clinical experts chosen?*

**Response:** We have now added an additional sentence to the Methods section detailing how workshop participants were selected (based on their previous and/or current interaction with the UK NSC).

***Question/Comment:*** *Lines 325-330. The base case results of this exploratory study showed that the modelled VCI-based and LLP-based pathways led to the detection of a greater proportion of VP pregnancies and a higher number of referrals to TVS than the no screening or IVF-based pathways. These higher VP detection rates also led to a correspondingly lower proportion of VP pregnancies resulting in perinatal death. The VCI-based pathway resulted in the highest VP detection rate (78.9%) and lowest proportion of perinatal death in VP pregnancies (14.2%); however, it also resulted in the detection of almost all VCI pregnancies, compared to minimal detection of VCI in the other pathways, and required a substantially higher number of additional TAS scans which, currently, are rarely recommended in practice.*

*-is TVS the mode of diagnosis, in which case why are additional TAS referenced?*

**Response:** As outlined in the clarification of the modelled screening strategies further above, TVS for VP does indeed represent the final, diagnostic step of each pathway. Additional scans in the form of TAS have also been considered as intermediate steps of the different pathways, in order to explore potential resource implications of the different strategies, for example where additional scans would be required over and above what is expected for current clinical practice (please also refer to our response to the corresponding query further below).

***Question/Comment:*** *Lines 356-360: This case-fatality rate is informed by the literature on clinically presenting VP and is also aligned with the conclusions made from a UK single-centre study by Zhang et al. where the authors estimated that around half of the 21 ultrasound-detected cases of VP (in a cohort of 26,830 pregnancies) would have resulted in stillbirth if they had not been diagnosed prenatally.[2, 20]*

*However, these estimates of VP-related mortality contrast with a 2017 national clinical surveillance study conducted in the UK where, in a cohort of approximately 750,000 pregnancies, six deaths to VP were reported.[18]*

*-It is true that the estimates of VP and mortality associated with it vary. The authors reference a UKOSS study (ref 18) which is an abstract and contains incomplete information. It is unusual that the UKOSS study on VP appears never to have been published, and this limits the robustness of the estimates given. Nevertheless UKOSS is a surveillance system and very likely under-estimates the true incidence of VP. The authors should comment on this point.*

**Response:** The manuscript offers variation in diagnostic criteria as a potential explanation for the differences between VP-related mortality estimates, and we consider this particularly plausible when comparing estimates derived from screening studies (such as Zhang et al.) and those from observation of clinically presenting VP (such as the UKOSS study).

Additional wording has now been added to acknowledge the possibility of under-reporting in the UKOSS study. However we consider it important to note, in the discussion with the Reviewer, that the UKOSS system is well known to UK obstetricians and that, when presenting clinically, VP is a condition which is difficult to miss; as such, we do not think that under-reporting should be overemphasised.

Further to this, and also in response to the Editor’s request above, we have included mention of the information from UKOSS being based on currently available, preliminary results, in order to acknowledge that the source of the information is indeed a conference abstract.

***Question/Comment:*** *Table 3: The premise that an additional TAS (presumably at 32 weeks) would be required in 862,785 women to screen for VCI need some explanation. Many UK units routinely report cord insertion-this takes around 30 seconds as part of the 18-20 week (or indeed 11-14 week) scan.*

**Response:** We can confirm that the model currently assumes that additional TAS for VCI would be required for all pregnancies, in addition to the routine fetal anomaly scan at 18^+0^ to 20^+6^ weeks, as, to our understanding, the detection of VCI is not formally mandated by the scan base menu detailed in the NHS fetal anomaly screening programme handbook, or in NICE or RCOG guidance.

However, we acknowledge that this is likely a conservative assumption based on the fact that some UK units may indeed routinely report cord insertion, and have added further wording to this effect in the Discussion.
